# Association between change in cardiovascular risk scores and future cardiovascular disease: analyses of data from the Whitehall II longitudinal, prospective cohort study

**DOI:** 10.1016/S2589-7500(21)00079-0

**Published:** 2021-07

**Authors:** Joni V Lindbohm, Pyry N Sipilä, Nina Mars, Anika Knüppel, Jaana Pentti, Solja T Nyberg, Philipp Frank, Sara Ahmadi-Abhari, Eric J Brunner, Martin J Shipley, Archana Singh-Manoux, Adam G Tabak, G David Batty, Mika Kivimäki

**Affiliations:** Clinicum, Department of Public Health, University of Helsinki, Helsinki, Finland; Department of Epidemiology and Public Health, University College London, London, UK; Clinicum, Department of Public Health, University of Helsinki, Helsinki, Finland; Helsinki Institute of Life Science, University of Helsinki, Helsinki, Finland; Institute for Molecular Medicine Finland, HiLIFE, University of Helsinki, Helsinki, Finland; Cancer Epidemiology Unit, Nuffield Department of Population Health, University of Oxford, Oxford, UK; Clinicum, Department of Public Health, University of Helsinki, Helsinki, Finland; Department of Public Health, University of Turku, Turku, Finland; Clinicum, Department of Public Health, University of Helsinki, Helsinki, Finland; Department of Epidemiology and Public Health, University College London, London, UK; Ageing Epidemiology Research Unit, Imperial College London, London, UK; Université de Paris, Inserm U1153, Epidemiology of Ageing and Neurodegenerative diseases, Paris, France; Department of Epidemiology and Public Health, University College London, London, UK; Department of Epidemiology and Public Health, University College London, London, UK; Department of Epidemiology and Public Health, University College London, London, UK; Department of Epidemiology and Public Health, University College London, London, UK; 1st Department of Medicine, Semmelweis University Faculty of Medicine, Budapest, Hungary; Department of Epidemiology and Public Health, University College London, London, UK; School of Biological and Population Health Sciences, Oregon State University, Corvallis, OR, USA; Clinicum, Department of Public Health, University of Helsinki, Helsinki, Finland; Department of Epidemiology and Public Health, University College London, London, UK

## Abstract

**Background:**

Evaluation of cardiovascular disease risk in primary care, which is recommended every 5 years in middle-aged and older adults (typical age range 40–75 years), is based on risk scores, such as the European Society of Cardiology Systematic Coronary Risk Evaluation (SCORE) and American College of Cardiology/American Heart Association Atherosclerotic Cardiovascular Disease (ASCVD) algorithms. This evaluation currently uses only the most recent risk factor assessment. We aimed to examine whether 5-year changes in SCORE and ASCVD risk scores are associated with future cardiovascular disease risk.

**Methods:**

We analysed data from the Whitehall II longitudinal, prospective cohort study for individuals with no history of stroke, myocardial infarction, coronary artery bypass graft, percutaneous coronary intervention, definite angina, heart failure, or peripheral artery disease. Participants underwent clinical examinations in 5-year intervals between Aug 7, 1991, and Dec 6, 2016, and were followed up for incident cardiovascular disease until Oct 2, 2019. Levels of, and 5-year changes in, cardiovascular disease risk were assessed using the SCORE and ASCVD risk scores and were analysed as predictors of cardiovascular disease. Harrell’s C index, continuous net reclassification improvement, the Akaike information criterion, and calibration analysis were used to assess whether incorporating change in risk scores into a model including only a single risk score assessment improved the predictive performance. We assessed the levels of, and 5-year changes in, SCORE and ASCVD risk scores as predictors of cardiovascular disease and disease-free life-years using Cox proportional hazards and flexible parametric survival models.

**Findings:**

7574 participants (5233 [69·1%] men, 2341 [30·9%] women) aged 40–75 years were included in analyses of risk score change between April 24, 1997, and Oct 2, 2019. During a mean follow-up of 18·7 years (SD 5·5), 1441 (19·0%; 1042 [72·3%] men and 399 [27·7%] women) participants developed cardiovascular disease. Adding 5-year change in risk score to a model that included only a single risk score assessment improved model performance according to Harrell’s C index (from 0·685 to 0·690, change 0·004 [95% CI 0·000 to 0·008] for SCORE; from 0·699 to 0·700, change 0·001 [0·000 to 0·003] for ASCVD), the Akaike information criterion (from 17 255 to 17 200, change −57 [95% CI −97 to −13] for SCORE; from 14 739 to 14 729, change −10 [−28 to 7] for ASCVD), and the continuous net reclassification index (0·353 [95% CI 0·234 to 0·447] for SCORE; 0·232 [0·030 to 0·344] for ASCVD). Both favourable and unfavourable changes in SCORE and ASCVD were associated with cardiovascular disease risk and disease-free life-years. The associations were seen in both sexes and all age groups up to the age of 75 years. At the age of 45 years, each 2-unit improvement in risk scores was associated with an additional 1·3 life-years (95% CI 0·4 to 2·2) free of cardiovascular disease for SCORE and an additional 0·9 life-years (95% CI 0·5 to 1·3) for ASCVD. At age 65 years, this same improvement was associated with an additional 0·4 life-years (95% CI 0·0 to 0·7) free of cardiovascular disease for SCORE and 0·3 life-years (95% CI 0·1 to 0·5) for ASCVD. These models were developed into an interactive calculator, which enables estimation of the number of cardiovascular disease-free life-years for an individual as a function of two risk score measurements.

**Interpretation:**

Changes in the SCORE and ASCVD risk scores over time inform cardiovascular disease risk prediction beyond a single risk score assessment. Repeat data might allow more accurate cardiovascular risk stratification and strengthen the evidence base for decisions on preventive interventions.

**Funding:**

UK Medical Research Council, British Heart Foundation, Wellcome Trust, and US National Institute on Aging.

## Introduction

Guidelines for cardiovascular disease prevention recommend assessment of an individual’s future risk to inform decisions on lifestyle and medical interventions.^1–3^ The risk of a cardiovascular disease event is computed using risk scores that require entry of data for multiple risk factors, commonly including age, sex, blood pressure, smoking, cholesterol, and diabetes status.^1–3^ Although various scores exist and risk thresholds vary between them, the basic principles of prevention are similar across published guidelines.^1–3^ Individuals at low risk are advised to maintain a healthy lifestyle to remain at low risk, whereas lifestyle changes are recommended and preventive medication (eg, statins or blood pressure-lowering medication) considered for those at borderline risk. For people identified as being at high risk, both preventive medication and lifestyle changes are indicated.

To facilitate monitoring of disease risk, physicians are recommended to reassess risk levels every 5 years in middle-aged and older adults (aged 40–75 years), with any decisions on preventive interventions based on only the latest risk factor measurement.^1–3^ However, lifestyle and medical interventions are predicated on modifying cardiovascular disease risk, and an earlier risk factor measurement can contain useful information about an individual’s risk history.^4^ Whether changes in these risk scores improve risk stratification relative to a single updated risk assessment is unknown. It is also unclear whether changes in risk scores are associated with cardiovascular disease-free life-years (a measure that might be more useful for risk communication than relative risk estimates) and whether these associations attenuate with increasing age.^5^ Better understanding of these issues might facilitate setting of individualised targets for risk factor levels in future health checks and take into account the effect of increasing age.

Accordingly, we aimed to assess whether changes in risk scores computed with the European Society of Cardiology’s Systematic Coronary Risk Evaluation (SCORE)^3^ and the American College of Cardiology/American Heart Association’s Atherosclerotic Cardiovascular Disease (ASCVD) risk algorithms^2^—the two most commonly used risk models—were associated with subsequent cardiovascular disease event rates and life-years free of cardiovascular disease, and whether any associations were affected by increasing age. On the basis of our results, we developed an interactive illustration-of-concept online tool to quantify the effect of changes in risk scores on life-years free of cardiovascular disease.

## Methods

### Study design and participants

We analysed data from the Whitehall II longitudinal, prospective cohort study, in which all civil servants (government employees) aged 35–55 years based in 20 departments in London, UK, were invited to participate between Sept 10, 1985, and March 29, 1988, and 10 308 (73·0%) of 14 121 agreed.^6^ In accordance with current guidelines,^1–3^ cardiovascular disease risk factors were assessed at 5-year intervals between: Aug 7, 1991, and May 10, 1993; April 24, 1997, and Jan 8, 1999; Oct 8, 2002, and Sept 10, 2004; Oct 10, 2007, and Nov 18, 2009; Jan 27, 2012, and Oct 30, 2013; and Feb 2, 2015, and Dec 6, 2016. Participants with no history of stroke, myocardial infarction, coronary artery bypass graft, percutaneous coronary intervention, definite angina, heart failure, or peripheral artery disease at baseline were eligible for inclusion in the analysis. As our outcomes were new (incident) cases of cardiovascular disease and life-years free of cardiovascular disease, individuals with prevalent cardiovascular disease were excluded from all analyses.

**Figure 1:**
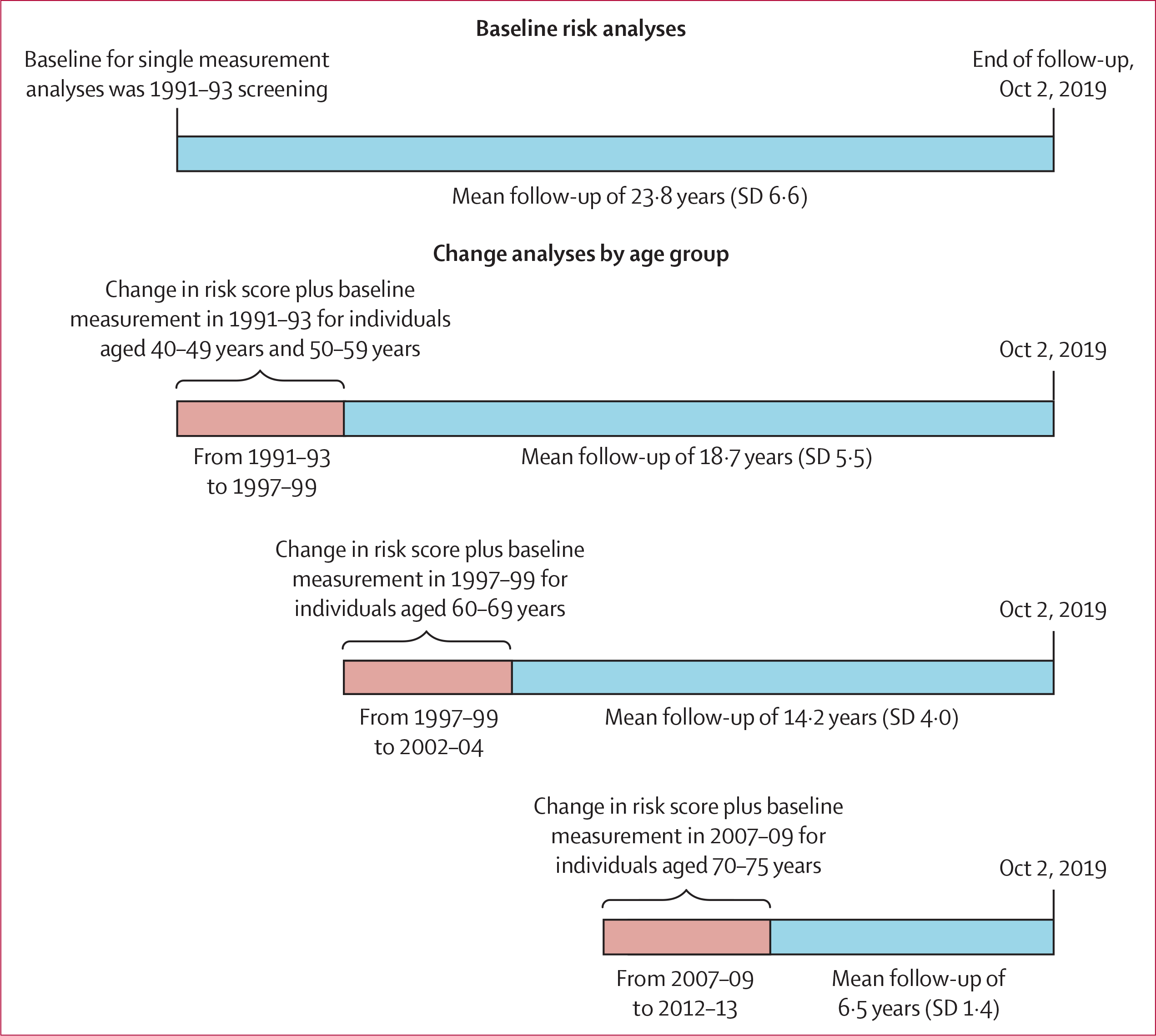
Study design Schematic showing analyses of baseline risk scores and changes in risk scores, by age group, and follow-up for cardiovascular disease events.

For analyses of changes in cardiovascular disease risk scores, we used the clinical assessments in 1991–93 and 1997–99 for individuals aged 40–59 years at baseline, change between 1997–99 and 2002–04 for individuals aged 60–69 years, and change between 2007–09 and 2012–13 for those aged 70–75 years (figure 1). In all analyses, cardiovascular disease event surveillance started after the second risk score assessment and ended on Oct 2, 2019. Research ethics approval was given by the University College London Hospital (London, UK) Committee on the Ethics of Human Research, and participants provided written informed consent at each examination.

### Data collection

Standard self-administered questionnaires provided data on age, sex, ethnicity, socioeconomic status (high, intermediate, or low), medication type, smoking, physical activity (ideal, intermediate, or poor), and diet (ideal, intermediate, or poor). Ideal physical activity was defined as 150 min or more moderate activity, 75 min or more vigorous activity, or a combination of moderate and vigorous activity for 150 min or more, per week; intermediate activity was defined as 1–149 min moderate activity, 1–74 min vigorous activity, or a combination of moderate and vigorous activity for 1–149 min or more, per week; and poor activity was defined as being physically inactive. Ideal diet was defined as four or more, intermediate as two or three, and poor as none or one of the following dietary items: 4·5 cups or more of fruit and vegetables per day, 3·5 or more 100 g servings of fish per week, three or more 85 g servings of fibre-rich foods per day, sodium consumption less than 1500 mg per day, and 450 kcal or less of sugar-sweetened beverages per week. At baseline, socioeconomic position was approximated by the British civil service occupational grade: a three-level variable representing high (administrative), intermediate (professional or executive), and low (clerical or support) grades. This measure is a comprehensive marker of socioeconomic circumstances and is related to salary, social status, level of responsibility at work, and future pension. Administrative grades in the British civil service represent the highest grades; administrators run the different government departments. Experienced clinical nurses measured height, weight, and systolic and diastolic blood pressure, and took blood samples for lipid and glucose assays.^6^ Additionally, participants were instructed to bring all of their medications to each 5-yearly clinical examination.

The SCORE and ASCVD risk scores were constructed using age, sex, total cholesterol, HDL cholesterol, systolic blood pressure, antihypertensive medication, smoking, and diabetes in accordance with the original descriptions (appendix 1 p 3).^2,3^ Higher scores denote higher risk. The thresholds for low, borderline, and high cardiovascular disease risk were derived from current guidelines: for SCORE, these corresponded to less than 1%, 1% to less than 5%, and 5% or more; and for ASCVD, these corresponded to less than 5%, 5% to less than 7·5%, and 7·5% or more.^2,3^ For SCORE, we used the algorithm for a low-risk population because our cohort originated in England, which is classified as a low-risk country according to the original SCORE report.^7^

WHO International Classification of Diseases (ICD) codes were retrieved from the National Health Service (NHS) Hospital Episode Statistics database records and from mortality registers using individual NHS identification numbers for linkage.^8^ The NHS provides nearly complete comprehensive health-care coverage for all individuals legally resident in the UK. Cardiovascular disease diagnoses, as ascertained via the Hospital Episode Statistics database, have shown 70% sensitivity and 96% specificity against standard biomedical examinations.^8^ Incident cardiovascular disease was denoted by stroke (ICD-10 codes under I60, I61, I63, and I64; ICD-9 codes 430, 431, 434, and 436), myocardial infarction (ICD-10 codes under I21; ICD-9 codes under 410), heart failure (ICD-10 codes under I50), definite angina (ICD-10 codes under I20; ICD-9 codes under 410, both verified from medical records), peripheral artery disease (ICD-10 codes I70.2, I73.3, I73.9, I74.3–5, E10.5, E11.5, E12.5, E13.5, and E14.5; ICD-9 codes 250.7, 440.2, 440.4, 443.8–9, 444.2, and 444.81), coronary artery bypass graft (self-reported), or percutaneous coronary intervention (self-reported).

### Statistical analysis

Participants were followed up until their first cardiovascular disease event, death, or the end of follow-up (Oct 2, 2019), whichever occurred first. To examine whether incorporating change in risk scores improved the predictive performance of the SCORE and ASCVD risk scores, we used Harrell’s C index, continuous net reclassification improvement, the Akaike information criterion (AIC), and calibration analysis.^9–11^ A smaller AIC value indicates better fit, and a difference of more than 10 units is considered to provide strong support for a better fit.^12^ We used an optimism index to quantify overfitting. Optimism in discrimination and calibration was estimated by drawing 200 repeated bootstrap samples (with replacement) from the original data.

We examined the associations of SCORE and ASCVD risk categories (low, borderline, and high) with cardiovascular disease-free life-years, defined as the number of years without cardiovascular disease up to the age of 90 years. In further analyses, we calculated the hazard ratios (HRs) and 95% CIs for the association of change in risk scores with cardiovascular disease-free life-years using flexible parametric survival models. Years free of cardiovascular disease were estimated with change in restricted mean survival times using change in risk score between the ages of 40 years and 75 years. All analyses on change in risk scores were adjusted for baseline cardiovascular disease risk, ethnicity, and socioeconomic status. We used flexible parametric survival models to estimate HRs and 95% CIs for the associations between risk scores and cardiovascular disease events. In all parametric analyses, we first built a Cox proportional hazards model and examined the survival curves, Schoenfeld residuals, and log–log plots to detect any violations in proportionality assumption, and the degrees of freedom needed for the restricted cubic spline function used for the baseline hazard rate and for the potential time-dependent effects. The final model was chosen using the AIC.^12^

We tested the robustness of our findings in several sensitivity analyses. To examine whether our findings were attributable to pharmacological interventions, we repeated the main analysis after excluding individuals who were taking or had initiated risk factor-modifying medication (ie, lipid-lowering, antihypertensive, antidiabetic, or anticoagulation medication) between the surveys. To address potential survival bias, we studied the effect of competing risk of death using Fine and Gray models.^13^ Owing to missing risk factor data, we used multiple imputation by chained equations to supplement missing values in change analyses (appendix 1 pp 4–10). The sensitivity to outcome definition was examined by including only major cardiovascular diseases (myocardial infarction and stroke) as the outcome.

We derived baseline survival and adjusted coefficients for change in risk for each risk score category between the ages of 40 years and 75 years (appendices 2–4) and then estimated changes in cardiovascular disease-free life-years as a function of changes in SCORE and ASCVD risk scores for continuous age. An extension incorporating this information was integrated into the SCORE and ASCVD risk scores and is available as an interactive online tool. This tool provides an estimate of gained or lost life-years when earlier risk factor measurement is taken into account in addition to the updated risk factor measurement (analysis of risk history), and estimated life-years free of cardiovascular disease in the next risk assessment as a function of anticipated risk factor levels at that time (analysis of targeted change) with accompanying information about how lifestyle changes recommended in current American College of Cardiology/American Heart Association and European Society of Cardiology guidelines^14–16^ change risk factor levels (appendix 1 p 11).

We used Stata (version 16.1 MP) and R (version 3.6.0) for statistical analyses and developed the online tool with R (version 3.6.0).

### Role of the funding source

The funders of the study had no role in study design, data collection, data analysis, data interpretation, or writing of the report.

## Results

**Figure 2:**
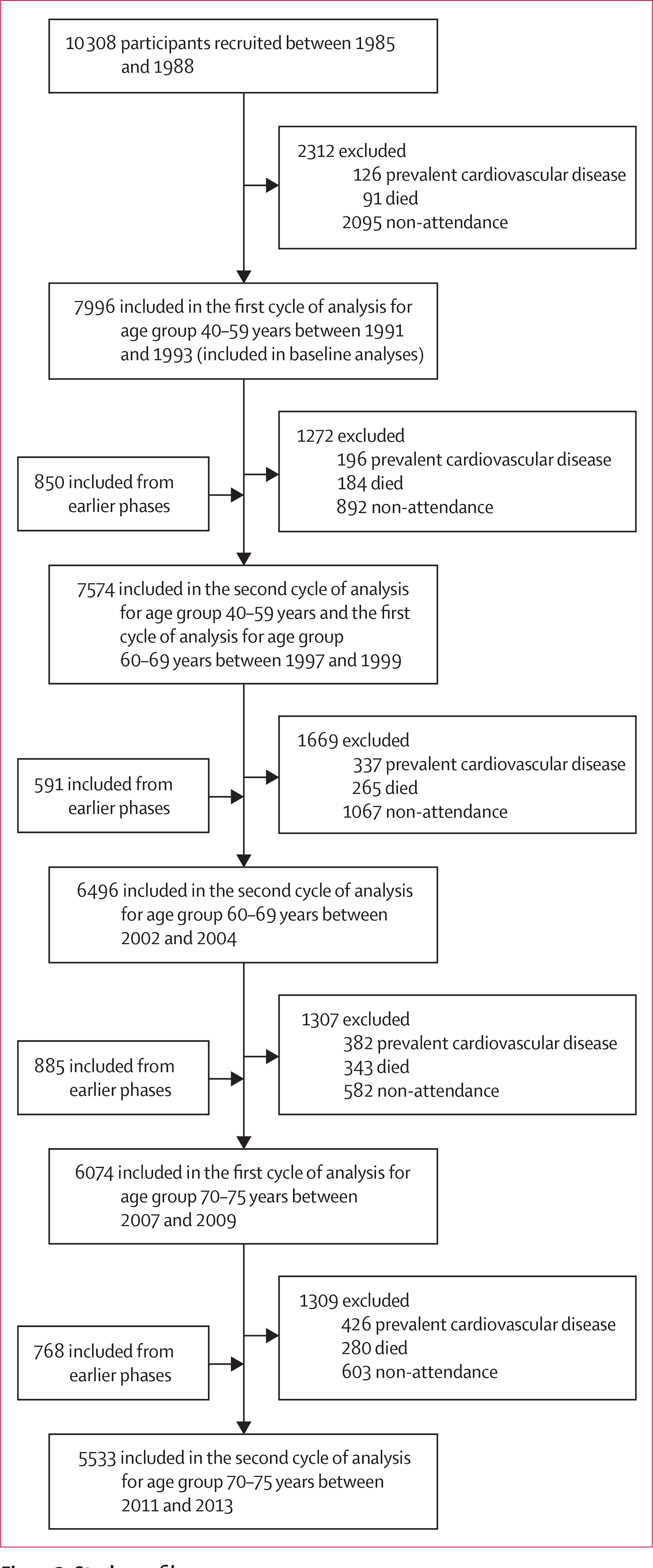
Study profile

7996 participants aged 40–63 years between Aug 7, 1991, and May 10, 1993, were included in the baseline analyses (figure 2). Both SCORE and ASCVD risk scores increased over time. This increase was coupled with an increase in systolic blood pressure, use of antihypertensive medication, body-mass index, diabetes, HDL cholesterol, and physical activity, and a decrease in smoking and total cholesterol (table 1). In risk score change analyses, after exclusion of 422 individuals with prevalent cardiovascular disease, those who died, and those who did not attend the clinical visits, 1441 (19·0%; 1042 [72·3%] men, 399 [27·7%] women) of 7574 people remaining in the study (5233 [69·1%] men, 2341 [30·9%] women) were diagnosed with incident cardiovascular disease during a mean follow-up of 18·7 years (SD 5·5). Of the diagnoses, 391 (27·1%) were for myocardial infarction, 288 (20·0%) for coronary artery intervention, 284 (19·7%) for stroke, 233 (16·2%) for definite angina, 173 (12·0%) for heart failure, and 72 (5·0%) for peripheral artery disease. The distribution of risk factors by outcome status after each 5-year survey is presented in appendix 1 (pp 12–16).

With cardiovascular disease as the outcome, adding change in risk scores to a model that included a single risk score from the first or a later survey improved Harrell’s C index (from 0·685 to 0·690, change 0·004 [95% CI 0·000 to 0·008] for SCORE; from 0·699 to 0·700, change 0·001 [0·000 to 0·003] for ASCVD), the AIC (from 17 255 to 17 200, change −57 [95% CI −97 to −13] for SCORE; from 14 739 to 14 729, change −10 [−28 to 7] for ASCVD), and the continuous net reclassification index (0·353 [95% CI 0·234 to 0·447] for SCORE; 0·232 [0·030 to 0·344] for ASCVD) for both SCORE and ASCVD (table 2). These models also had acceptable calibration and were not over-optimistic in terms of discrimination (appendix 1 pp 17–18).

A decrease in continuous SCORE and ASCVD variables over 5 years was associated with lower cardiovascular disease risk, whereas an increase was associated with higher risk (appendix 1 pp 19–21). Change in risk scores were related to greater changes in cardiovascular disease risk in women and in younger participants (p<0·0001 for both scores). For example, a 2-unit decrease in risk scores among participants aged 40–49 years was associated with a 24% decrease in cardiovascular disease risk for SCORE and 19% decrease for ASCVD, compared with reductions of 7% and 6%, respectively, for those aged 60–69 years.

**Figure 3:**
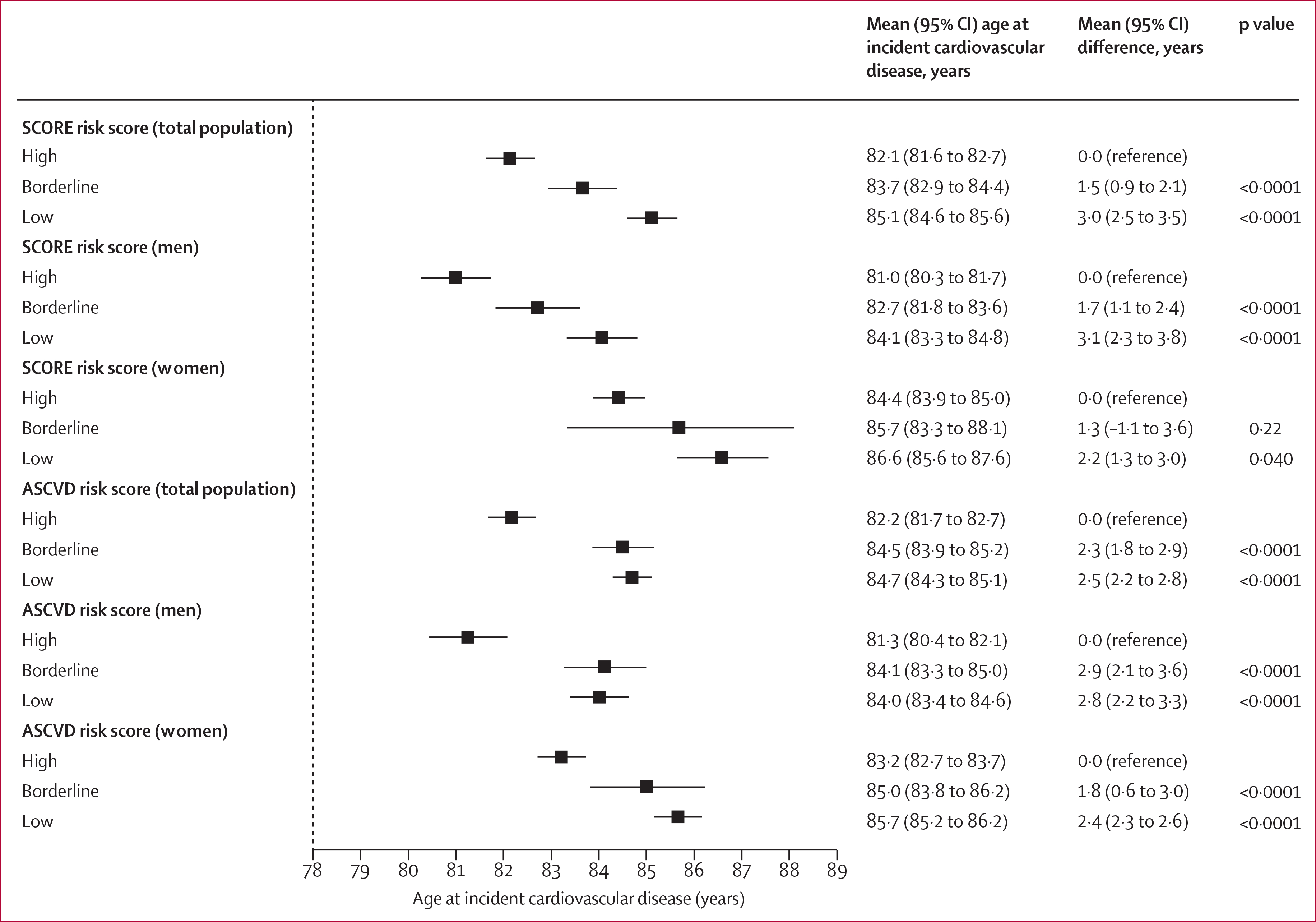
Estimated age at first cardiovascular disease event by risk category and sex All analyses were adjusted for socioeconomic status and ethnicity. The mean restricted survival time was estimated between the ages of 40 and 90 years. ASCVD=American College of Cardiology/American Heart Association Atherosclerotic Cardiovascular Disease. SCORE=European Society of Cardiology Systematic Coronary Risk Evaluation.

Baseline cardiovascular disease risk measured with SCORE and ASCVD was related to cardiovascular disease-free life-years (figure 3). Compared with people in the high-risk category, people with borderline levels of risk factors at 40 years of age had an additional 1·3–2·9 cardiovascular disease-free life-years and those with low levels of risk factors had an additional 2·2–3·1 cardiovascular disease-free life-years.

**Figure 4:**
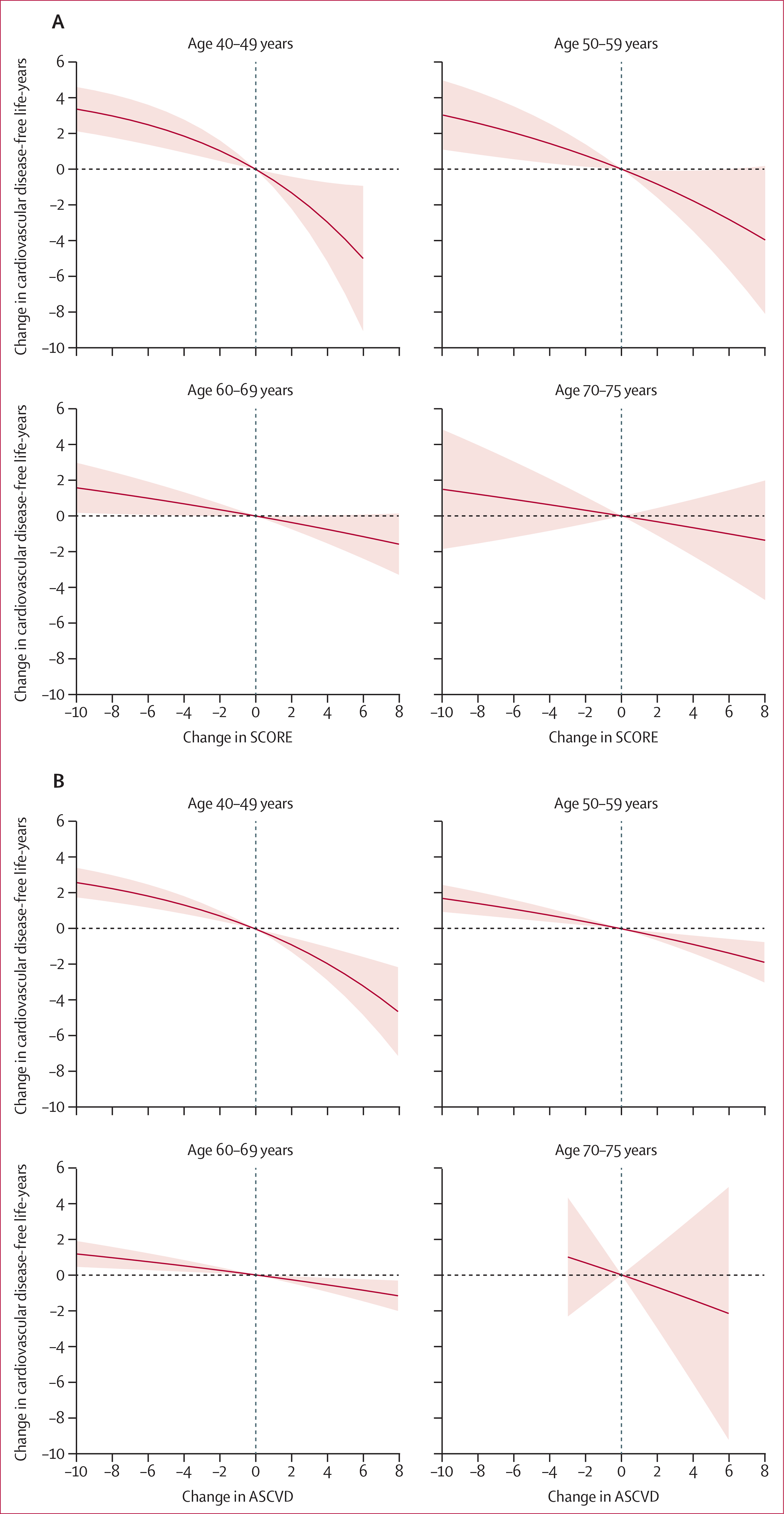
Association between cardiovascular disease-free life-years and change in cardiovascular risk scores, by age group European Society of Cardiology Systematic Coronary Risk Evaluation (SCORE; A) and American College of Cardiology/American Heart Association Atherosclerotic Cardiovascular Disease (ASCVD; B) risk score. The shaded area shows the 95% CI. All analyses were adjusted for socioeconomic status, ethnicity, and baseline risk; no change is the reference. p value for age by risk score interaction is <0·0001 for both scores.

Changes in risk scores were also associated with changes in the number of disease-free life-years (figure 4). Each 2-unit improvement in risk scores at 45 years of age was associated with an increase of 1·3 life-years (95% CI 0·4–2·2) free of cardiovascular disease for SCORE and 0·9 life-years (0·5–1·3) for ASCVD, compared with an increase of 0·4 life-years (0·0–0·7) and 0·3 life-years (0·1–0·5), respectively, for the same improvement at 65 years of age. These results did not change in analyses including only the study participants who changed their SCORE or ASCVD score without preventive medication, analyses including only major cardiovascular diseases (ie, myocardial infarction and stroke) as the outcome, or in analyses taking into account the competing risk of death (appendix 1 pp 22–23, 25). Between 4% and 28% of participants had missing data for at least one risk factor used to construct the scores; however, multiple imputation of missing data did not materially change the results (appendix 1 p 24).

We developed our predictive model into an online extension to the SCORE and ASCVD risk algorithms. This tool enables estimation of the number of cardiovascular disease-free life-years for an individual as a function of two risk score measurements. This interactive calculator incorporating analyses of risk history and targeted change is available online (appendix 1 pp 26–27).

## Discussion

In our analyses of longitudinal data from the Whitehall II prospective cohort study, we found that, relative to a single cardiovascular risk score, using risk scores computed from repeat measurements made at 5-year intervals improved the predictive performance of two widely used cardiovascular risk scores—SCORE and ASCVD. Change in risk scores was associated with incident cardiovascular disease events and life-years free of cardiovascular disease in both men and women and in all age groups up to the age of 75 years, although this association was strongest in younger individuals. For example, each 2-unit improvement in the SCORE and ASCVD risk scores at the age of 45 years was associated with an additional 1 year of cardiovascular disease-free life. These findings were not sensitive to competing risks, missing data, or inclusion of specific cardiovascular disease outcomes, and the results were replicated in individuals who improved their SCORE and ASCVD risk scores without medication.

Previous studies have shown associations between increases in a specific risk factor and progression of atherosclerosis.^4^ To our knowledge, this is the first long-term study to quantify the extent to which changes in commonly used cardiovascular disease risk scores improve prediction of incident cardiovascular disease and disease-free life-years beyond the baseline measurement. Models using information about change in risk scores were superior to models including only baseline measurements with the three performance metrics used (Harrell’s C index, continuous net reclassification improvement, and the AIC). The improvement persisted when change was measured using individual patient history (ie, previous 5-year measurement) and when change was modelled based on the subsequent 5-year measurement.

Both increases and decreases in risk scores were associated with cardiovascular disease-free life-years, and these associations were stronger in younger individuals. The explanation for this age interaction might lie in the higher cumulative burden of atherosclerosis in older individuals.^17^ Subclinical atherosclerosis develops long before the onset of major cardiovascular disease, and the longer the atherosclerotic processes are uncontrolled, the less opportunities there are for effective preventive interventions. This hypothesis is supported by studies showing that the effects of interventions such as smoking cessation and antihypertensive or statin medication tend to attenuate at older ages.^18–20^

We chose to use the SCORE and ASCVD risk scores because they are widely used risk scores in primary prevention. Compared with associations with the ASCVD risk score, the associations were somewhat stronger but less accurate with SCORE. The greater HR per unit change in SCORE might be due to the narrower range in SCORE, as it is designed to use fatal cardiovascular diseases as outcomes. The greater predictive accuracy of the ASCVD score, in turn, might be due to the greater number of variables and interaction terms included. For example, the favourable effects of smoking cessation and lipid lowering among older individuals have been captured in the ASCVD risk score with interaction terms between age and smoking and between age and cholesterol. A further source of accuracy in the ASCVD score might be the wider age range in the derivation cohort^2,3^ and use of both fatal or non-fatal major cardiovascular disease outcomes (ie, myocardial infarction and stroke). We chose to use a broad combination of incident non-fatal and fatal cardiovascular diseases (myocardial infarction, coronary heart disease, stroke, heart failure, and peripheral artery disease) as our primary outcome because knowledge of prevention of any of these diseases would best serve the patient. Furthermore, the main findings were replicated using an alternative outcome that included only myocardial infarction and stroke.

Our results persisted among individuals who achieved risk reduction without cardiovascular disease medication. This finding suggests that monitoring changes in the SCORE and ASCVD risk scores might provide valuable information about the effectiveness of lifestyle changes. The change in risk score is an objective metric that captures the net effect of all lifestyle modifications resulting in changes in risk factors in addition to increasing age between risk assessments. We observed that clinically meaningful improvement in the risk scores would require substantial changes in lifestyle, such as quitting smoking, or a decrease in systolic blood pressure or total cholesterol similar to that achieved by low-intensity antihypertensive or statin therapy. These results indicate that commonly recommended lifestyle interventions might be insufficient to effectively prevent or delay the onset of cardiovascular events in most cases. This idea is supported by the results of intensive multifactorial lifestyle intervention studies, which show only a minor improvement in risk factors and no decrease in the incidence of cardiovascular disease in the intervention group.^21,22^

Our findings support the hypothesis that measuring changes in risk scores can supplement current approaches such as those suggested by the Million Hearts initiative^23^ and the LIFE time perspective Cardio-Vascular Disease model,^24^ which are based on known intervention effects. When applied to an individual aged 65 years with a 10% cardiovascular disease risk, these approaches assume that initiation of statin therapy and lowering of LDL cholesterol by 1 mmol/L would lower cardiovascular disease risk by a factor of 0·78 (ie, the risk would decrease from 10·0% to 7·8%), which is roughly the intervention effect of statins reported in randomised controlled trials.^23^ This approach is appealing as it would also allow modelling of age, sex, and race-specific intervention effects if reliable randomised controlled trials for these subgroups exist. However, the approach has not been validated in intervention studies, does not aid in recommendation of a combination of lifestyle changes, and cannot be applied when no estimate of the effect of intervention exists as is the case when multiple lifestyle changes are combined. In these situations, measuring changes in risk scores could provide an alternative.

Our study has some important strengths, including the long follow-up and repeated standardised risk factor measurements, which enabled us to examine change in risk over time. Additionally, we were able to quantify the association between changes in cardiovascular disease risk scores and the number of cardiovascular disease-free life-years—a metric that might be more intuitive than relative risk. We reported Harrell’s C index and continuous net reclassification index, which are commonly used predictive performance metrics in the medical literature; however, these measures have some limitations. Harrell’s C index is not optimal for within-study comparisons, when superior ranking is not observed across all risk score values or there are different misclassification costs for different classifiers.^25^ The continuous net reclassification index lacks a clinically meaningful scale for the purposes of translation. However, it is reassuring that both the Harrell’s C index and the continuous net reclassification index provide results that are consistent with our AIC statistics, indicating improved predictive performance for models including repeat measurements. We also observed acceptable calibration and very minor overfitting, which supports the validity of the change model.

The findings of our study should be interpreted with the following caveats. First, Whitehall II is an occupational cohort that included participants who were healthier than the general population. This means that the incidence of disease and prevalence of risk factors are likely to be an underestimation of those in the general population. However, previously reported associations between risk factors and cardiovascular disease in this cohort have been in agreement with those observed in the general population,^26^ and the changes in cardiovascular disease risk factors that we observed during follow-up were similar to those reported in studies in community-based cohorts.^27^ Second, cardiovascular disease ascertainment in the Whitehall II cohort was based on linked electronic health records, which has high specificity, but moderate sensitivity.^8^ However, this is not a source of major bias in assessing associations between risk factors and disease. Accordingly, electronic health records have been used in the UK to develop risk prediction models that have been externally validated and are currently used in clinical practice.^28,29^ Third, despite a high response rate at each survey (range 78–95%), accumulating loss to follow-up might have biased our estimates. However, our imputed analyses and recent studies suggest that estimates of cardiovascular disease risk and risk progression in this cohort change little after taking missing data into account.^30^ Fourth, we were unable to externally validate our results; further cohort studies with 5-yearly risk factor measurements are therefore needed to confirm our findings. Fifth, the use of blood pressure-lowering medication was self-reported by study participants. This might have led to some misclassifications and potentially biased our estimates of the association between change in risk scores and cardiovascular diseases. However, assessment of antihypertensive medication use with questionnaires has been reported to have higher sensitivity (0·95 [95% CI 0·93–0·96]) and specificity (0·97 [0·96–0·98]) when validated with health insurance claims.^31^ Additionally, when we excluded participants taking any preventive cardiovascular medication our results were essentially unchanged.

In conclusion, this study provides evidence from a well characterised cohort of middle-aged and older-aged UK men and women initially free of cardiovascular disease that monitoring changes in risk scores, such as SCORE and ASCVD, might have predictive and clinical utility. For the purposes of future translational research on the benefits of expanding current risk assessment to incorporate multiple risk assessments, we developed an interactive calculator to quantify life-years gained or lost by changes in risk scores. This tool can be used to validate our findings and to examine in clinical practice whether use of risk assessment tools based on repeat measurements is practical, improves risk communication between general practitioners and patients, and informs setting of realistic target goals for lifestyle and pharmacological interventions.

## Supplementary Material

Appendix 1

Appendix 2

Appendix 3

Appendix 4

## Figures and Tables

**Table 1:** Characteristics of study participants

	Risk factor assessment 1991–93 (n=7996)	Risk factor assessment 1997–99 (n=7574)	Risk factor assessment 2002–04 (n=6496)	Risk factor assessment 2007–09 (n=6074)	Risk factor assessment 2011–13 (n=5533)

**Risk score components**					
Age, years	50·0 (6·0)	55·6 (6·0)	60·9 (5·9)	65·6 (5·8)	69·2 (5·7)
Sex					
Men	5532 (69·2%)	5233 (69·1%)	4526 (697%)	4210 (69·3%)	3847 (69·5%)
Women	2464 (30·8%)	2341 (30·9%)	1970 (30·3%)	1864 (30·7%)	1686 (30·5%)
Ethnicity					
White	7212/7966 (90·5%)	6933/7565 (91·6%)	5992/6483 (92·4%)	5623/6060 (92·8%)	5155/5526 (93·3%)
Other	754/7966 (9·5%)	632/7565 (8·4%)	491/6483 (7·6%)	437/6060 (7·2%)	371/5526 (6·7%)
Diabetes	155 (1·9%)	271 (3·6%)	388 (6·0%)	523 (8·6%)	541 (9·8%)
Smokers	1060/7820 (13·6%)	735/6965 (10·6%)	472/6458 (7·3%)	372/5924 (6·3%)	185/5423 (3·4%)
Antihypertensive medication	503 (6·3%)	941 (12·4%)	1419 (21·8%)	1969/6064 (32·5%)	2143/5531 (38·7%)
Systolic blood pressure, mm Hg	121 (14)	123 (17)	128 (17)	126 (16)	128 (17)
Total cholesterol, mmol/L	6·5 (1·2)	5·9 (1·1)	5·8 (1·0)	5·3 (1·1)	5·2 (1·1)
HDL cholesterol, mmol/L	1·4 (0·4)	1·5 (0·4)	1·6 (0·5)	1·6 (0·5)	1·7 (0·5)
Other risk factors					
Body-mass index, kg/m^2^	25·3 (37)	26·2 (4·0)	26·7 (4·4)	26·8 (4·5)	26·7 (4·5)
Physical activity					
Poor	1374/7823 (17·6%)	877/6886 (12·7%)	517/6338 (8·2%)	518/5958 (8·7%)	493/5454 (9·0%)
Intermediate	3008/7823 (38·5%)	2607/6886 (37·9%)	2351/6338 (37·1%)	2173/5958 (36·5%)	2002/5454 (36·7%)
Ideal	3441/7823 (44·0%)	3402/6886 (49·4%)	3470/6338 (54·7%)	3267/5958 (54·8%)	2959/5454 (54·3%)
Diet					
Poor	2229/7846 (28·4%)	1289/5244 (24·6%)	1301/5322 (24·4%)	1065/4886 (21·8%)	958/4441 (21·6%)
Intermediate	4954/7846 (63·1%)	3438/5244 (65·6%)	3500/5322 (65·8%)	3328/4886 (68·1%)	3025/4441 (68·1%)
Ideal	663/7846 (8·5%)	517/5244 (9·9%)	521/5322 (9·8%)	493/4886 (10·1%)	458/4441 (10·3%)
Socioeconomic status					
Low	1537 (19·2%)	1388 (18·3%)	1067 (16·4%)	974 (16·0%)	843 (15·2%)
Intermediate	3954 (49·4%)	3731 (49·3%)	3251 (50·0%)	3049 (50·2%)	2795 (50·5%)
High	2505 (31·3%)	2455 (32·4%)	2178 (33·5%)	2051 (33·8%)	1895 (34·2%)
**SCORE risk score**					
Risk calculation, %	1·2% (1·2)	2·0% (1·9)	3·3% (2·8)	4·1% (3·1)	5·4% (3·8)
Risk categories					
High (≥5%)	103/7747 (1·3%)	388/6152 (6·3%)	1056/5931 (17·8%)	1429/5385 (26·5%)	1957/4770 (41·0%)
Borderline (1% to <5%)	3113/7747 (40·2%)	3552/6152 (57·7%)	4117/5931 (69·4%)	3701/5385 (68·7%)	2779/4770 (58·3%)
Low (<1%)	4531/7747 (58·5%)	2212/6152 (36·0%)	758/5931 (12·8%)	255/5385 (4·7%)	34/4770 (0·7%)
**ASCVD risk score**					
Risk calculation, %	4·9% (4·4)	6·7% (5·5)	10·1% (7·6)	13·4% (9·0)	18·1% (11·5)
Risk categories					
High (≥7·5%)	1626/7722 (21·1%)	1913/5457 (35·1%)	3213/5930 (54·2%)	3802/5381 (70·7%)	4039/4770 (84·7%)
Borderline (5% to <7·5%)	1157/7722 (15·0%)	929/5457 (17·0%)	1091/5930 (18·4%)	745/5381 (13·8%)	396/4770 (8·3%)
Low (<5%)	4939/7722 (64·0%)	2615/5457 (47·9%)	1626/5930 (27·4%)	834/5381 (15·5%)	335/4770 (7·0%)

Data are mean (SD), n (%), or n/N (%). This analysis includes individuals who participated in both 1991–93 and 1995–97, or 1995–97 and 2002–04, or 2005–07 and 2011–13 assessments pairs.

Some percentages do not add up to 100% because of rounding. ASCVD=American College of Cardiology/American Heart Association Atherosclerotic Cardiovascular Disease. SCORE=European Society of Cardiology Systematic Coronary Risk Evaluation.

**Table 2:** Harrell’s C index, Akaike information criterion, and net reclassification index before and after adding information on change in risk score to a model including a single risk score measurement*

	Harrell’s C index	Akaike information criterion	Net reclassification index
**Analysis of risk history**			
SCORE risk score			
Baseline at the risk factor measurement	0·685 (reference)	17 255	··
Baseline measurement + change from the preceding risk factor measurement	0·690	17 200	··
Change (95% CI)†	0·004 (0·000 to 0·008)	−57 (−97 to −13)†	··
Optimism in derivation sample‡	0·002	··	··
Reclassification in cases (95% CI)	··	··	−0·006 (−0·060 to 0·063)
Reclassification in non-cases (95% CI)	··	··	0·360 (0·248 to 0·411)
Continuous net reclassification index (95% CI)	··	··	0·353 (0·234 to 0·447)
ASCVD risk score	··	··	··
Baseline at the risk factor measurement	0·699 (reference)	14 739 (reference)	··
Baseline measurement + change from the preceding risk factor measurement	0·700	14 729	··
Change (95% CI)†	0·001 (0·000 to 0·003)	−10 (−28 to 7)†	··
Optimism in derivation sample‡	0·002	··	··
Reclassification in cases (95% CI)	··	··	−0·101 (−0·156 to −0·027)
Reclassification in non-cases (95% CI)	··	··	0·333 (0·110 to 0·442)
Continuous net reclassification index (95% CI)	··	··	0·232 (0·030 to 0·344)
**Analysis of targeted change**	··	··	··
SCORE risk score	··	··	··
Baseline at the first risk factor measurement	0·684 (reference)	17 229	··
Baseline measurement + change in the following risk factor measurement	0·690	17 200	··
Change (95% CI)†	0·006 (0·001 to 0·107)	−29 (−56 to −1)†	··
Optimism in derivation sample‡	0·002	··	··
Reclassification in cases (95% CI)	··	··	−0·085 (−0·137 to −0·010)
Reclassification in non-cases (95% CI)	··	··	0·469 (0·271 to 0·496)
Continuous net reclassification index (95% CI)	··	··	0·384 (0·219 to 0·445)
ASCVD risk score	··	··	··
Baseline at the risk factor measurement	0·690 (reference)	14 775 (reference)	··
Baseline measurement plus change in the following risk factor measurement	0·700	14 729	··
Change (95% CI)†	0·010 (0·004 to 0·015)	−47 (−75 to −19)†	··
Optimism in derivation sample‡	0·002	··	··
Reclassification in cases (95% CI)	··	··	−0·156 (−0·215 to −0·071)
Reclassification in non-cases (95% CI)	··	··	0·534 (0·424 to 0·566)
Continuous net reclassification index (95% CI)	··	··	0·379 (0·226 to 0·475)

ASCVD=American Heart Association Atherosclerotic Cardiovascular Disease. SCORE=European Society of Cardiology Systematic Coronary Risk Evaluation.

* All analyses were adjusted for the baseline risk (measured either in 1991–93 or 1997–99), socioeconomic status, and ethnicity. Follow-up for incident cardiovascular disease starts at later measurement in prospective and retrospective change analysis.

† A decrease of 10 units or more in Akaike information criterion is considered strong evidence of better fit.

‡ Optimism index describes overfitting of the final model compared with 200 bootstrap samples with replacement.

## Research in context

### Evidence before this study

Quantifying an individual’s probability of developing cardiovascular disease using a risk score is central to disease prevention in middle-aged and older-aged populations (typical age range 40–75 years). Current prevention guidelines recommend health checks every 5 years to evaluate risk and, if needed, appropriate lifestyle interventions are recommended and drug therapies prescribed. Since lifestyle and medical interventions alter cardiovascular disease risk, changes in risk assessment scores following interventions could potentially be used to improve risk stratification compared with a single updated risk assessment alone, as well as to set targets for such interventions. We searched PubMed for research articles, with no language restriction, published up to Oct 30, 2020, using the following search terms: “cardiovascular disease”, “myocardial infarction”, “risk score”, “risk algorithm”, “change”, “improvement”, “reduction”, and “decline”, but found no studies examining the extent to which changes in cardiovascular risk scores are associated with changes in the number of cardiovascular disease-free life-years, or any studies examining an association between changes in risk scores and cardiovascular disease events.

### Added value of this study

In this longitudinal, prospective cohort study of UK adults, 5-year changes in cardiovascular risk scores were associated with future incidence of cardiovascular disease. Both favourable and unfavourable 5-year changes in the European Society of Cardiology Systematic Coronary Risk Evaluation (SCORE) and the American College of Cardiology/American Heart Association Atherosclerotic Cardiovascular Disease (ASCVD) risk scores predicted incident cardiovascular disease events and life-years free of cardiovascular disease. These findings were seen in analyses taking into account competing risk of death, and were observed in both men and women and in different age groups up to the age of 75 years, with the strongest associations apparent in participants aged 40–49 years. For both the SCORE and ASCVD risk scores, adding 5-year change in risk score to a model that included a single risk score assessment improved predictive performance. To facilitate translational research on the use of repeated risk factor measurements in health care, our predictive model was developed into an online extension to the SCORE and ASCVD risk scores, which enables estimation of the number of cardiovascular disease-free life-years for an individual as a function of two risk score measurements.

### Implications of all the available evidence

Risk assessment using the SCORE or ASCVD risk scores has traditionally been based on a single updated measurement of risk factors. In this longitudinal study of UK adults, 5-year changes in SCORE and ASCVD risk scores improved predictive performance for all age groups up to the age of 75 years. Future studies should examine the validity and generalisability of these findings in other populations and test in clinical practice whether use of risk assessment tools based on repeat measurements is practical, improves risk communication between general practitioners (primary care or family doctors) and patients, and informs setting of realistic target goals for lifestyle and pharmacological interventions. Our interactive online calculator will help to facilitate such translational research.
