# Supplementary material for: Association between change in cardiovascular risk scores and future cardiovascular disease: analyses of data from the Whitehall II longitudinal, prospective cohort study
Source: Lancet Digit Health. Author manuscript; Available in PMC 2021 Sep 27. (PMC8474012; doi:10.1016/S2589-7500(21)00079-0)
Supplement: Appendix 1 [file NIHMS1719402-supplement-Appendix_1.pdf]

THE LANCET
Digital Health

Supplementary appendix

This appendix formed part of the original submission and has been peer reviewed. We post it as supplied by the authors.

Supplement to: Lindbohm JV, Sipilä PN, Mars N, et al. Association between change in cardiovascular risk scores and future cardiovascular disease: analyses of data from the Whitehall II longitudinal, prospective cohort study. *Lancet Digit Health* 2021; **3:** e434–44.

APPENDIX

Contents **Page**

Contents	Page
**Appendix Table 1.** Characteristics of data comprising the ASCVD and SCORE risk algorithms	3
**Appendix Table 2.** Missing value patterns of risk algorithm components in phase 1991-1993, age, sex and antihypertensive medication variables had no missing values.	4
**Appendix Table 3.** Missing value patterns of risk algorithm components in phase 1997-1999, age, sex and antihypertensive medication variables had no missing values.	5
**Appendix Table 4.** Missing value patterns of risk algorithm components in phase 2002-2004, age, sex and antihypertensive medication variables had no missing values.	6
**Appendix Table 5.** Missing value patterns of risk algorithm components in phase 2007-2009, age, and sex variables had no missing values.	7
**Appendix Table 6.** Missing value patterns of risk algorithm components in phase 2011-2013, age, and sex variables had no missing values.	8
**Appendix Methods, Statistical analysis**	9-10
**Appendix Table 7.** Intervention effects for change in systolic blood pressure and total cholesterol summarized from articles referenced in AHA and ESC primary prevention guidelines	11
**Appendix Table 8**. Risk factor values in those who did and did not develop cardiovascular diseases after baseline measurement in 1991/1993	12
**Appendix Table 9**. Risk factor values in those who did and did not develop cardiovascular diseases after baseline measurement in 1997/1999.	13
**Appendix Table 10**. Risk factor values in those who did and did not develop cardiovascular diseases after baseline measurement in 2002/2004	14
**Appendix Table 11**. Risk factor values in those who did and did not develop cardiovascular diseases after baseline measurement in 2007/2009	15
**Appendix Table 12**. Risk factor values in those who did and did not develop cardiovascular diseases after baseline measurement in 2011/2013	16
**Appendix Figure 1.** Calibration plot for a model including change and baseline measurement of SCORE algorithm	17

Appendix Figure 2. Calibration plot for a model including change and baseline measurement of ASCVD algorithm**.** 18

Appendix Figure 3. Hazard ratio and 95% confidence intervals for CVD risk according to 5-year change in SCORE (part A) and ASCVD (part B) algorithms (no change is the reference) by age group. 19-20

Appendix Figure 4. Hazard ratios for incident cardiovascular disease per two-unit improvement in SCORE and ASCVD algorithm over 5-years stratified by age and sex 21

Appendix Figure 5. Hazard ratios for incident cardiovascular disease per two-unit improvement in SCORE and ASCVD algorithm over 5-years stratified by age and sex from model including only those who achieved the change without medication 22

Appendix Figure 6. Hazard ratios for incident cardiovascular disease per two-unit improvement in SCORE and ASCVD algorithm over 5-years stratified by age and sex from model including competing risk of death. 23

Appendix Figure 7. Hazard ratios for incident cardiovascular disease per two-unit improvement in SCORE and ASCVD algorithm over 5-years stratified by age and sex from multiple imputation analysis 24

Appendix Figure 8. Hazard ratios for incident cardiovascular disease per two-unit improvement in SCORE and ASCVD algorithm over 5-years stratified by age and sex from model including only myocardial infarctions and strokes as outcome. 25

Online tool and examples 26-27

References 28-30

Appendix Table 1. Characteristics of data comprising the ASCVD and SCORE risk algorithms

	**ASCVD**	**SCORE**
Participants	4 pooled prospective studies	12 prospective studies
Time period	1968-1993	1972-1991
Population	Black and white Americans	Europeans from 11 countries
Sample size	205178 (57% men)	24626 (48% men)
Baseline age range	20-79	40-65
Variables included	Age, sex, race (white or other/African American), total cholesterol, HDL-C, SBP, antihypertensive treatment, DM, smoking + interaction terms when appropriate	Sex, age, total cholesterol, SBP, smoking status.
Outcomes	Nonfatal myocardial infarction or coronary heart disease death or fatal or nonfatal stroke	Fatal following diseases: hypertension, hypertensive heart disease, hypertensive chronic kidney disease, hypertensive heart disease, acute venous embolism or thrombosis of deep vessels of lower extremity, myocardial infarction, angina pectoris, ischemic heart disease, conduction disorders, cardiac dysrhythmias, heart failure, intracranial haemorrhage, ischemic stroke, transient cerebral ischemia, atherosclerosis, aortic aneurysm and dissection, peripheral vascular disease, death within 24 h of symptom onset (71% of all events were coronary heart diseases)

Appendix Table 2. Missing value patterns of risk algorithm components in phase 1991-1993, age, sex and antihypertensive medication variables had no missing values.
1 = observed, 0 = missing data

Systolic blood pressure	Ethnicity	Total cholesterol	High-density lipoprotein cholesterol	Smoking	Prevalence (%)
1	1	1	1	1	96
0	1	1	1	0	2
1	0	1	1	1	<1
1	1	1	0	1	<1
1	1	0	0	0	<1
0	1	1	1	1	<1
0	1	0	0	0	<1
0	1	0	0	1	<1
1	0	1	1	0	<1
1	0	0	0	0	<1

Appendix Table 3. Missing value patterns of risk algorithm components in phase 1997-1999, age, sex and antihypertensive medication variables had no missing values.
1 = observed, 0 = missing data

Systolic blood pressure	Ethnicity	Total cholesterol	High-density lipoprotein cholesterol	Smoking	Prevalence (%)
1	1	1	1	1	72
0	1	0	0	1	10
1	1	1	0	1	9
0	1	0	0	0	7
1	1	0	0	1	<1
1	1	1	1	0	<1
0	1	1	1	1	<1
0	0	0	0	0	<1
1	0	1	1	1	<1
1	1	1	0	0	<1
1	1	0	1	1	<1
1	1	0	0	0	<1
0	1	1	0	1	<1

Appendix Table 4. Missing value patterns of risk algorithm components in phase 2002-2004, age, sex and antihypertensive medication variables had no missing values.
1 = observed; 0 = missing data

Ethnicity	Smoking	Systolic blood pressure	Total cholesterol	High-density lipoprotein cholesterol	Prevalence (%)
1	1	1	1	1	91
1	1	0	0	0	7
1	1	1	0	0	2
1	0	1	1	1	<1
0	1	1	1	1	<1
1	0	0	0	0	<1
0	1	0	0	0	<1
1	1	0	1	1	<1
1	0	1	0	0	<1
1	1	1	1	0	<1

Appendix Table 5. Missing value patterns of risk algorithm components in phase 2007-2009, age, and sex variables had no missing values.
1 = observed, 0 = missing data

Antihypertensive medication	Ethnicity	Smoking	Systolic blood pressure	Total cholesterol	High-density lipoprotein cholesterol	Prevalence (%)
1	1	1	1	1	1	88
1	1	1	0	0	0	7
1	1	1	1	0	0	2
1	1	0	1	1	1	1
1	1	0	0	0	0	<1
1	1	0	1	0	0	<1
1	1	1	0	1	1	<1
1	1	1	0	1	1	<1
0	1	0	1	1	1	<1
0	1	1	1	1	1	<1
1	0	1	0	0	0	<1
0	1	0	0	0	0	<1
0	1	0	1	0	0	<1
0	1	1	0	0	0	<1
1	0	1	1	0	0	<1

Appendix Table 6. Missing value patterns of risk algorithm components in phase 2011-2013, age, and sex variables had no missing values.
1 = observed, 0 = missing data

Antihypertensive medication	Ethnicity	Smoking	Systolic blood pressure	Total cholesterol	High-density lipoprotein cholesterol	Prevalence (%)
1	1	1	1	1	1	86
1	1	1	0	0	0	9
1	1	1	1	0	0	2
1	1	0	1	1	1	1
1	1	0	0	0	0	<1
1	1	0	1	0	0	<1
1	1	1	0	1	1	<1
1	0	1	1	1	1	<1
1	0	1	0	0	0	<1
0	1	0	1	1	1	<1
0	1	1	0	0	0	<1

Appendix Methods

Statistical analysis

Participants were followed until their first cardiovascular disease event, death, or the end of follow-up (October 2nd 2019), whichever occurred first. To examine whether incorporating change in risk scores improved the predictive performance of SCORE and ASCVD, we used Harrell's C-index, continuous net reclassification improvement (NRI), Akaike Information Criterion (AIC), and calibration analysis.[1–3] Harrell's C-index and continuous NRI are commonly used in the medical literature as predictive performance metrics (higher values indicate better risk stratification). The C-index directly quantifies movement of predicted individual risk when a new variable is introduced into a prediction model.[2] Unlike categorical NRI, continuous NRI is not influenced by the correct scaling of the model and thus can be used for inter-study comparison.[2] AIC measures how well a model fits with the dataset: it penalizes models that use more variables. Smaller AIC indicates better fit and difference over 10 units is considered as strong support for better fit.[4] We used an optimism index to quantify overfitting. Optimism in discrimination and calibration was estimated by drawing 200 repeated bootstrap samples (with replacement) from the original data.

We examined the associations of SCORE and ASCVD risk categories (low, borderline, high) with cardiovascular disease-free life-years defined as the number of years aged without cardiovascular disease up to age 90. Further analyses characterised the association of change in risk scores with cardiovascular disease-free life-years. Years free of cardiovascular disease were estimated with change in restricted mean survival times and was estimated by using change in risk score between the ages of 40 and 75. All the analyses on change in risk scores were adjusted for baseline cardiovascular disease risk, ethnicity, and socioeconomic status. We used flexible parametric survival models to estimate hazard ratios (HRs) and 95% confidence intervals (CIs) for the associations between risk scores and cardiovascular disease events. In all parametric analyses, we first built a Cox proportional hazards model[5] and examined the survival curves, Schoenfeld residuals, and log-log plots to detect any violations in proportionality assumption, and degrees of freedom needed for the restricted cubic spline function used for the baseline hazard rate and for the potential time-dependent effects. The final model was chosen using AIC.[6]

We tested the robustness of our findings in several sensitivity analyses. Owing to missing risk factor data (4% to 28% had at least one risk factor missing), we used multiple imputation by chained equations to supplement missing values in change analyses.[7,8] In addition to repeated measurements of the CVD risk algorithm and their individual components, our imputation model included the Nelson-Aalen estimator, outcome data, socioeconomic status, ethnic origin (white or non-white), and repeated measurements of alcohol consumption, family history of myocardial infarction or stroke (in either parent or any sibling), and a 30-question General Health Questionnaire. The change variables were imputed using predictive mean matching with 5 nearest neighbours. We imputed the data in wide form to take into account the clustering of repeated measurements within individuals. The diagnostics of our imputation model suggested that 20 iterations and 30 imputations were sufficient for reproducible results. We then used flexible parametric survival models to estimate the hazard ratios with accompanying 95% confidence intervals for CVD risk algorithms and CVD events in each imputed dataset.[9] These models were summed up with Rubin’s rules to derive the final estimate.[10,11]

To address potential survival bias, we studied the effect of competing risk of death using Fine and Gray models.[12] To examine whether our findings were attributable to pharmacological interventions, we repeated the main analysis after excluding individuals who were taking or who had just initiated risk factor-modifying medication (i.e., lipid-lowering, antihypertensive, antidiabetic, or anticoagulation medication) between the surveys. The sensitivity

to outcome definition was examined by including only major cardiovascular diseases (myocardial infarction and stroke) as the outcome.

We derived baseline survival and adjusted coefficients for change in risk for each risk score category between ages 40 and 75 and then estimated changes in CVD-free life-years as a function of changes in SCORE and ASCVD risk scores for continuous age. An extension incorporating this information was integrated in the SCORE and ASCVD risk scores and is available as an interactive **Online tool**. The tool is divided to two parts. First part provides an estimate of gained or lost life-years when earlier risk factor measurement is taken into account in addition to the most updated risk factor measurement (analysis of risk history). The second part estimates life-years free of cardiovascular disease in the next risk assessment as a function of anticipated risk factor levels at that time (analysis of targeted change). The second part uses information about how lifestyle changes recommended in current AHA/ACC and ESC guidelines[13–15] change risk factor levels based on previous studies.[15–32] The aim of this part is to provide additional information for treatment decisions by illustrating how planned interventions, in combination, would alter current risk scores and the estimate of cardiovascular disease-free life-years at the time of the next risk assessment. The link for Online tool Github page is: https://github.com/ninamars/Change-in-CVD-risk-scores.

We used statistical software Stata (version 16·1 MP; Stata Corp, College Station, TX, USA) and R (version 3.6.0) in our analyses.

Appendix Table 12. Intervention effects for change in systolic blood pressure and total cholesterol summarized from articles referenced in AHA and ESC primary prevention guidelines* [13–32]

Intervention	Change in systolic blood pressure (mmHg)	Change in total cholesterol (mmol/L)
Diet		
Reduce 4·4g salt per day[13]	-5·4	-
Start DASH diet[14]	-11·4	-0·21
Increase dietary potassium to 3·5-5·0g per day[15]	-5·3	-
Reduce alcohol intake to ≤2 (men) and ≤1 (women) drinkd per day[16]	-3·1	-
Start Mediterranean diet[17]	-2·6	-0·4
Increase whole grain intake by 100g per day[18]	-	-0·2
Reduce dietary cholesterol by over 500mg per day[19]	-	-0·3
Reduce dietary cholesterol by over 900mg per day[19]	-	-0·5
Increase plant sterols/stanols 2g a day[20]	-	-0·4
Increase polyunsaturated fat intake by 10% of energy consumption[21]	-	-0·8
Start red yeast rice 1·2 g per day (includes lovastatin)[22]	-	-1
Physical activity		
Over 360 minute physical activity counselling[23]	-1·6	-0·1
Dynamic resistance training (3 sessions per week)[24,25]	-4·3	-0·1
Aerobic endurance training (3 sessions per week)[26.27]	-8·3	-0·1
Isometric training (3 sessions per week)[24,25]	-5·2	-0·1
Weight loss		
Per 5 kilograms reduction in weight[28]	-5·5	-0·5
Medication for hypertension[29,31]		
Monotherapy	-10	-
Combination therapy	-20	-
Statin medication[30,31,32] (Reduction in %)		
Low intensity	-	-11
Moderate intensity	-	-21
High intensity	-	-35

*For whole grain and red yeast rice consumption the dose per day is weighted average from doses used in studies meta-analysed. For statin therapy decrease in total cholesterol was estimated by assuming that LDL-C accounts 70% of total cholesterol and the reductions with low, moderate, and high intensity statin therapy are 15%, 30%, and 50%.

Appendix Table 7. Risk factor values in those who did and did not develop cardiovascular diseases after baseline measurement in 1991/1993.

	**No CVD**	**CVD**
**Number of participants (%)**	6328 (79·2)	1668 (20·8)
**Age, mean (SD)**	49·3 (5·9)	52·6 (5·9)
**Sex (Men), n (%)**	4301 (68·0)	1231 (73·8)
**Ethnicity (White), n (%)**	5795 (91·9)	1417 (85·2)
**Diabetes (Yes), n (%)**	78 (1·2)	77 (4·6)
**Smoking, n (%)**		
Non-smoker, n (%)	5447 (87·8)	1313 (81·1)
Current smoker, n (%)	754 (12·2)	306 (18·9)
**Antihypertensive medication use (Yes), n (%)**	291 (4·6)	212 (12·7)
**Systolic blood pressure (mmHg), mean (SD)**	120 (13)	124 (14)
**Total cholesterol (mmol/L), mean (SD)**	6·4 (1·1)	6·7 (1·2)
**HDL-C (mmol/L), mean (SD)**	1·5 (0·4)	1·3 (0·4)
**BMI (kg/m2), mean (SD)**	25·1 (3·6)	26·2 (4·2)
**Physical activity, n (%)**		
Inactive, n (%)	1014 (16·4)	360 (22·2)
Intermediate, n (%)	2452 (39·5)	556 (34·3)
High, n (%)	2737 (44·1)	704 (43·5)
**Diet, n (%)**		
Poor, n (%)	1758 (28·3)	471 (28·8)
Intermediate, n (%)	3943 (63·5)	1011 (61·9)
Ideal, n (%)	511 (8·2)	152 (9·3)
**Socioeconomic status, n (%)**		
Low, n (%)	1158 (18·3)	379 (22·7)
Intermediate, n (%)	3144 (49·7)	810 (48·6)
High, n (%)	2026 (32·0)	479 (28·7)
**SCORE, mean (SD)**	1·0 (1·0)	1·8 (1·4)
**ASCVD, mean (SD)**	4·2 (3·8)	7·4 (5·6)

Appendix Table 8. Risk factor values in those who did and did not develop cardiovascular diseases after baseline measurement in 1997/1999.

	**No CVD**	**CVD**
**Number of participants (%)**	6133 (81·0)	1441 (19·0)
**Age, mean (SD)**	55·0 (5·8)	58·5 (5·9)
**Sex (Men), n (%)**	4191 (68·3)	1042 (72·3)
**Ethnicity (White), n (%)**	5690 (92·9)	1243 (86·4)
**Diabetes (Yes), n (%)**	158 (2·6)	113 (7·8)
**Smoking, n (%)**		
Non-smoker, n (%)	5123 (90·5)	1107 (84·8)
Current smoker, n (%)	537 (9·5)	198 (15·2)
**Antihypertensive medication use (Yes), n (%)**	625 (10·2)	316 (21·9)
**Systolic blood pressure (mmHg), mean (SD)**	122 (16)	128 (17)
**Total cholesterol (mmol/L), mean (SD)**	5·9 (1·1)	6·0 (1·0)
**HDL-C (mmol/L), mean (SD)**	1·5 (0·4)	1·4 (0·4)
**BMI (kg/m2), mean (SD)**	26·0 (3·9)	26·9 (4·2)
**Physical activity, n (%)**		
Inactive, n (%)	693 (12·3)	184 (14·4)
Intermediate, n (%)	2146 (38·3)	458 (35·8)
High, n (%)	2766 (49·3)	636 (49·8)
**Diet, n (%)**		
Poor, n (%)	1058 (24·7)	231 (24·0)
Intermediate, n (%)	2805 (65·5)	633 (65·7)
Ideal, n (%)	418 (9·8)	99 (10·3)
**Socioeconomic status, n (%)**		
Low, n (%)	1063 (17·3)	325 (22·5)
Intermediate, n (%)	3046 (49·7)	685 (47·5)
High, n (%)	2024 (33·0)	431 (29·9)
**SCORE, mean (SD)**	1·8 (1·7)	2·9 (2·2)
**ASCVD, mean (SD)**	6·1 (5·0)	9·9 (6·4)

Appendix Table 9. Risk factor values in those who did and did not develop cardiovascular diseases after baseline measurement in 2002/2004.

	**No CVD**	**CVD**
**Number of participants (%)**	5465 (84·1)	1031 (15·9)
**Age, mean (SD)**	60·4 (5·8)	63·9 (5·9)
**Sex (Men), n (%)**	3769 (69·0)	757 (73·4)
**Ethnicity (White), n (%)**	5093 (93·3)	899 (87·5)
**Diabetes (Yes), n (%)**	270 (4·9)	118 (11·4)
**Smoking, n (%)**		
Non-smoker, n (%)	5079 (93·5)	907 (88·6)
Current smoker, n (%)	355 (6·5)	117 (11·4)
**Antihypertensive medication use (Yes), n (%)**	1050 (19·2)	369 (35·8)
**Systolic blood pressure (mmHg), mean (SD)**	127 (17)	132 (18)
**Total cholesterol (mmol/L), mean (SD)**	5·8 (1·0)	5·7 (1·1)
**HDL-C (mmol/L), mean (SD)**	1·6 (0·5)	1·5 (0·4)
**BMI (kg/m2), mean (SD)**	26·5 (4·3)	27·4 (4·7)
**Physical activity, n (%)**		
Inactive, n (%)	419 (7·8)	99 (9·9)
Intermediate, n (%)	1971 (36·9)	380 (38·2)
High, n (%)	2953 (55·3)	517 (51·9)
**Diet, n (%)**		
Poor, n (%)	1108 (24·6)	193 (23·5)
Intermediate, n (%)	2946 (65·5)	554 (67·5)
Ideal, n (%)	447 (9·9)	74 (9·0)
**Socioeconomic status, n (%)**		
Low, n (%)	854 (15·6)	213 (20·7)
Intermediate, n (%)	2758 (50·5)	493 (47·8)
High, n (%)	1853 (33·9)	325 (31·5)
**SCORE, mean (SD)**	3·0 (2·5)	4·5 (3·3)
**ASCVD, mean (SD)**	9·3 (6·8)	14·3 (8·8)

Appendix Table 10. Risk factor values in those who did and did not develop cardiovascular diseases after baseline measurement in 2007/2009.

	**No CVD**	**CVD**
**Number of participants (%)**	5378 (88·5)	696 (11·5)
**Age, mean (SD)**	65·1 (5·7)	68·8 (5·8)
**Sex (Men), n (%)**	3721 (69·2)	489 (70·3)
**Ethnicity (White), n (%)**	5011 (93·4)	612 (88·2)
**Diabetes (Yes), n (%)**	421 (7·8)	102 (14·7)
**Smoking, n (%)**		
Non-smoker, n (%)	4940 (94·0)	612 (91·2)
Current smoker, n (%)	313 (6·0)	59 (8·8)
**Antihypertensive medication use (Yes), n (%)**	1613 (30·0)	356 (51·1)
**Systolic blood pressure (mmHg), mean (SD)**	125 (16)	129 (17)
**Total cholesterol (mmol/L), mean (SD)**	5·3 (1·1)	5·1 (1·1)
**HDL-C (mmol/L), mean (SD)**	1·6 (0·5)	1·6 (0·4)
**BMI (kg/m2), mean (SD)**	26·6 (4·4)	27·5 (5·0)
**Physical activity, n (%)**		
Inactive, n (%)	449 (8·5)	69 (10·3)
Intermediate, n (%)	1912 (36·2)	261 (38·8)
High, n (%)	2924 (55·1)	343 (51·0)
**Diet, n (%)**		
Poor, n (%)	936 (21·5)	129 (24·3)
Intermediate, n (%)	2986 (68·6)	342 (64·5)
Ideal, n (%)	434 (10·0)	59 (11·1)
**Socioeconomic status, n (%)**		
Low, n (%)	829 (15·4)	145 (20·8)
Intermediate, n (%)	2705 (50·3)	344 (49·4)
High, n (%)	1844 (34·3)	207 (29·7)
**SCORE, mean (SD)**	3·9 (2·9)	5·3 (3·3)
**ASCVD, mean (SD)**	12·6 (8·5)	18·6 (10·4)

Appendix Table 11. Risk factor values in those who did and did not develop cardiovascular diseases after baseline measurement in 2011/2013.

	**No CVD**	**CVD**
**Number of participants (%)**	5085 (91·9)	448 (8·1)
**Age, mean (SD)**	69·0 (5·6)	72·8 (5·8)
**Sex (Men), n (%)**	3523 (69·3)	324 (72·3)
**Ethnicity (White), n (%)**	4758 (93·7)	397 (88·6)
**Diabetes (Yes), n (%)**	457 (9·0)	84 (18·8)
**Smoking, n (%)**		
Non-smoker, n (%)	4831 (96·8)	407 (94·7)
Current smoker, n (%)	162 (3·2)	23 (5·3)
**Antihypertensive medication use (Yes), n (%)**	1882 (37·0)	261 (58·3)
**Systolic blood pressure (mmHg), mean (SD)**	128 (16)	130 (18)
**Total cholesterol (mmol/L), mean (SD)**	5·2 (1·1)	5·0 (1·0)
**HDL-C (mmol/L), mean (SD)**	1·7 (0·5)	1·6 (0·5)
**BMI (kg/m2), mean (SD)**	26·6 (4·4)	27·3 (4·9)
**Physical activity, n (%)**		
Inactive, n (%)	428 (8·5)	65 (15·1)
Intermediate, n (%)	1830 (36·5)	172 (39·7)
High, n (%)	2763 (55·0)	196 (45·3)
**Diet, n (%)**		
Poor, n (%)	876 (21·4)	82 (23·6)
Intermediate, n (%)	2802 (68·5)	223 (64·1)
Ideal, n (%)	415 (10·1)	43 (12·4)
**Socioeconomic status, n (%)**		
Low, n (%)	755 (14·9)	88 (19·6)
Intermediate, n (%)	2571 (50·6)	224 (50·0)
High, n (%)	1759 (34·6)	136 (30·4)
**SCORE, mean (SD)**	5·1 (3·4)	7·2 (4·3)
**ASCVD, mean (SD)**	17·0 (10·7)	25·7 (13·3)

Appendix Figure 1. Calibration plot for a model including change and baseline measurement of SCORE algorithm**.** Red line is for model performance and black line quantifies overfitting of the model using 200 bootstrap samples with replacement. Blue bars describe distribution of predicted survival.

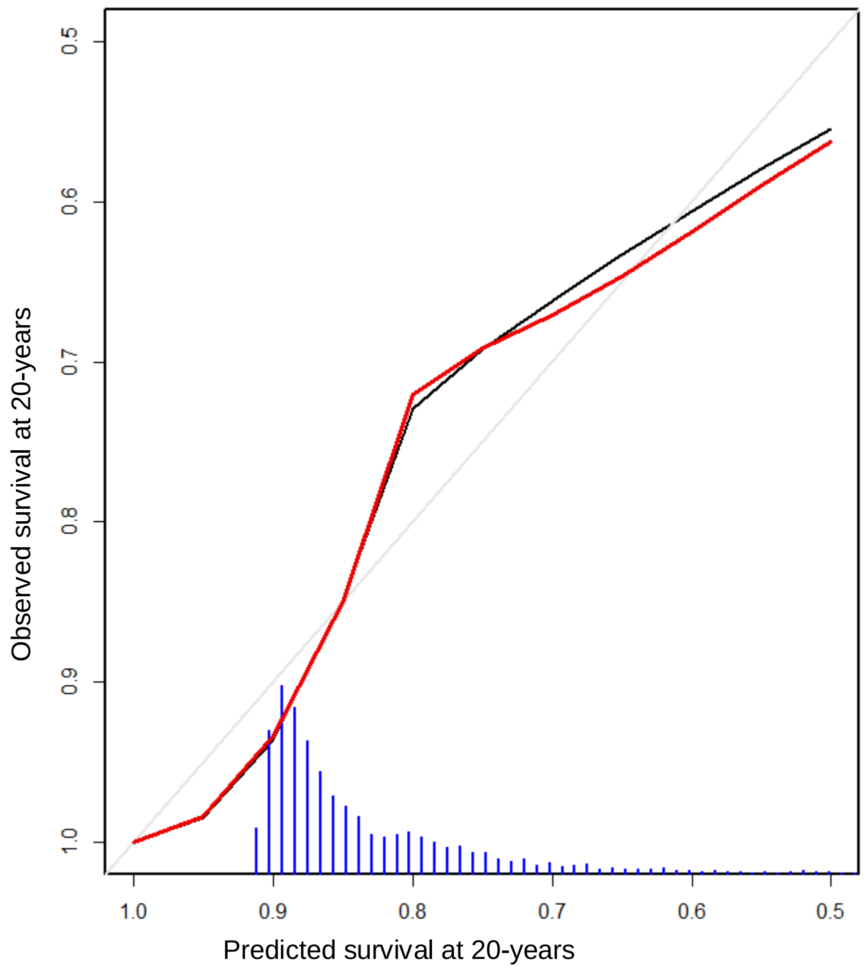

Appendix Figure 2. Calibration plot for a model including change and baseline measurement of ASCVD algorithm**.** Red line is for model performance and black line quantifies overfitting of the model using 200 bootstrap samples with replacement. Blue bars describe distribution of predicted survival.

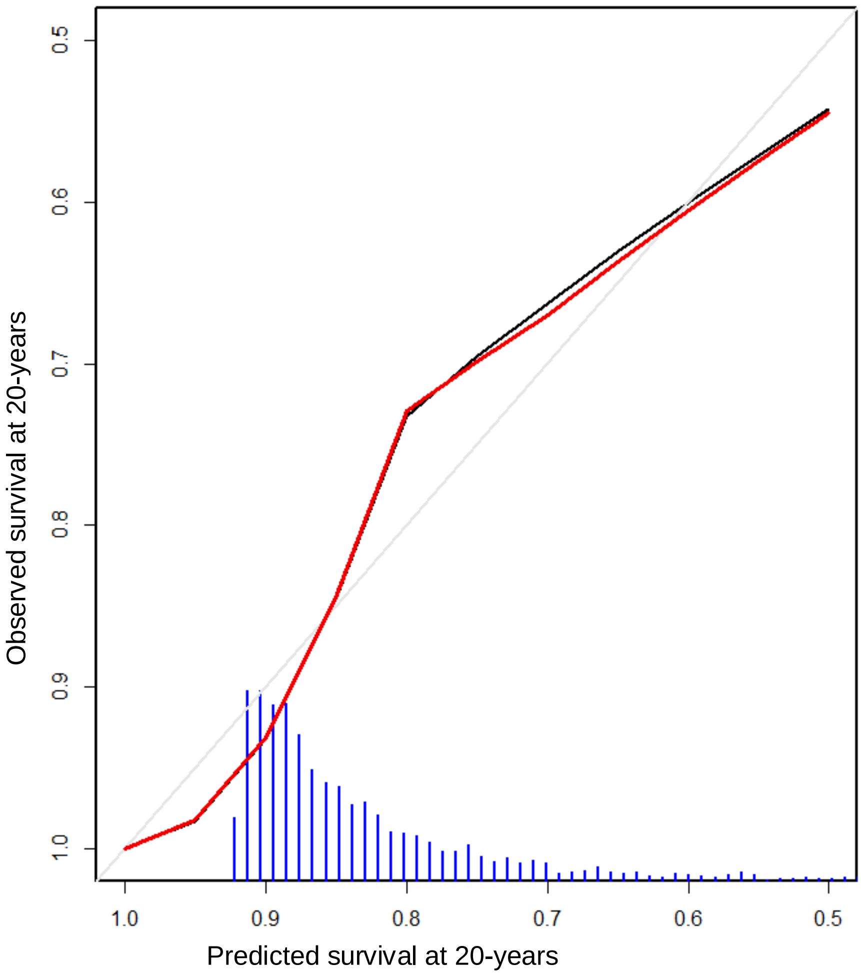

Appendix Figure 3. Hazard ratio and 95% confidence intervals for CVD risk according to 5-year change in SCORE (part A) and ASCVD (part B) algorithms (no change is the reference) by age group.*

A. The SCORE algorithm

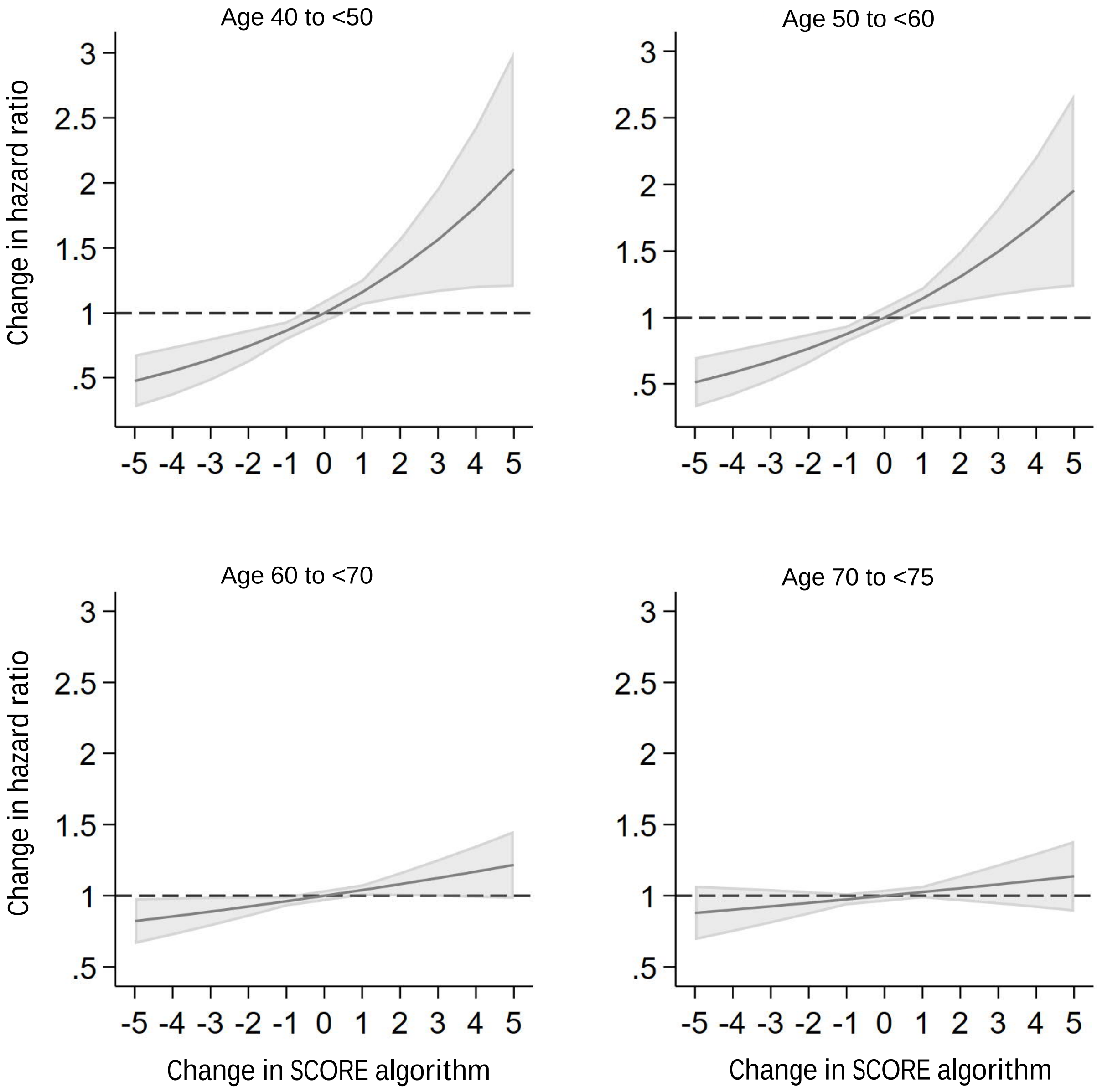

*All the analyses were adjusted for socioeconomic status, ethnicity, and baseline risk and p-value for age interaction is <0·0001. The analysis design is shown in Figure 1.

B. The ASCVD algorithm

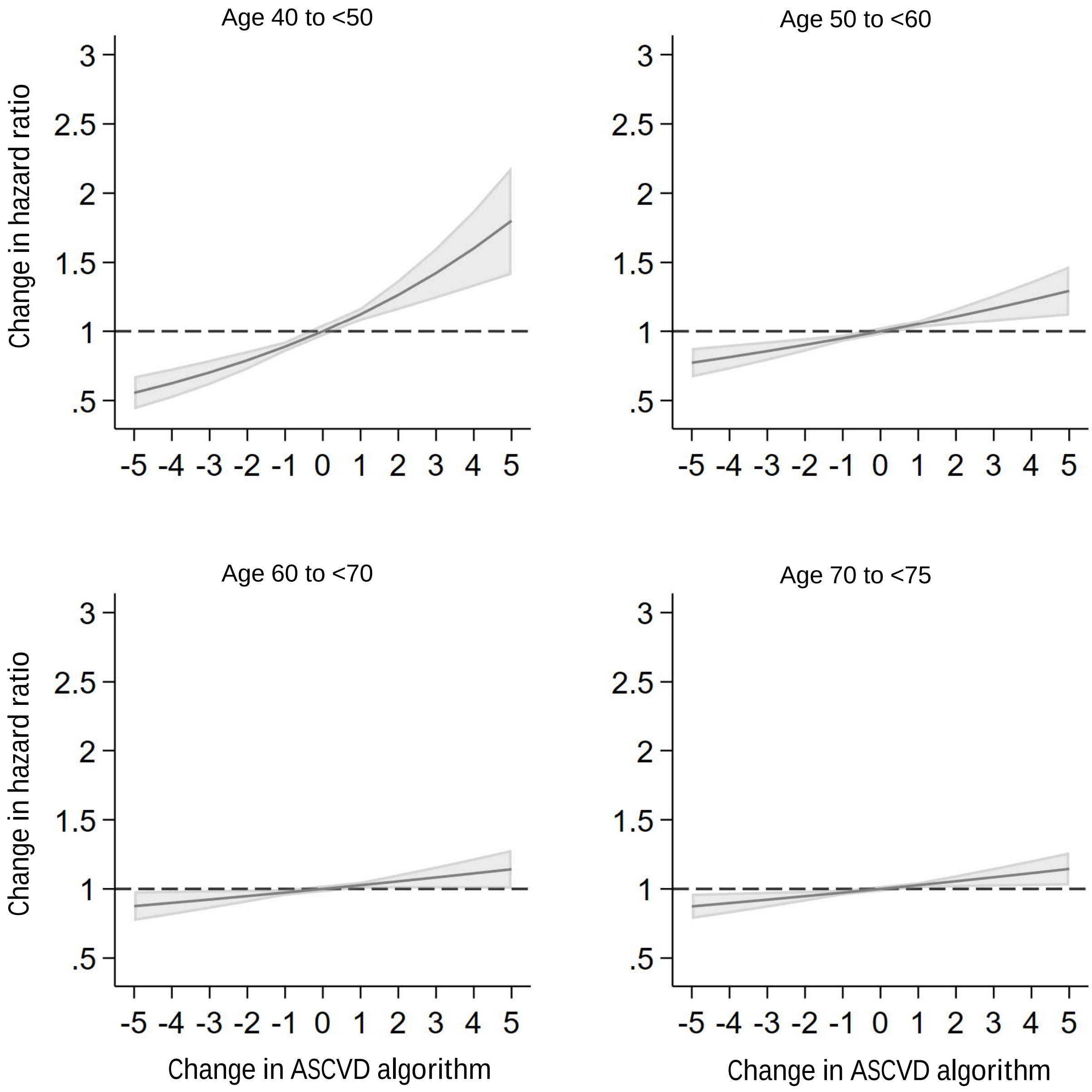

*All the analyses were adjusted for socioeconomic status, ethnicity, and baseline risk and p-value for age interaction is <0·0001. The analysis design is shown in Figure 1.

Appendix Figure 4. Hazard ratios for incident cardiovascular disease per two-unit improvement in SCORE and ASCVD algorithm over 5-years stratified by age and sex.

Risk score	Population	Hazard ratio per 2-unit improvement in risk score (95% confidence interval)	p-value
**SCORE**	All	0·80 (0·74 to 0·86)	<0·0001
	Men	0·84 (0·77 to 0·91)	<0·0001
	Women	0·65 (0·52 to 0·82)	0·0003
	40 to <50	0·76 (0·65 to 0·89)	0·0007
	50 to <60	0·87 (0·77 to 0·98)	0·0250
	60 to <70	0·93 (0·87 to 0·99)	0·0243
	70 to <75	0·94 (0·84 to 1·06)	0·3067
**ASCVD**	All	0·88 (0·85 to 0·91)	<0·0001
	Men	0·91 (0·88 to 0·95)	<0·0001
	Women	0·74 (0·69 to 0·80)	<0·0001
	40 to <50	0·81 (0·75 to 0·87)	<0·0001
	50 to <60	0·91 (0·86 to 0·95)	<0·0001
	60 to <70	0·94 (0·90 to 0·97)	0·0006
	70 to <75	0·93 (0·88 to 0·98)	0·0051

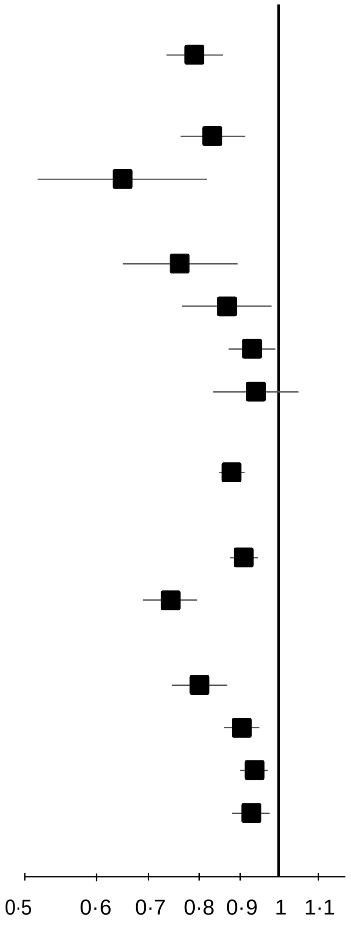

*All the analyses were adjusted for socioeconomic status, ethnicity, and baseline risk. Whitehall II included repeated screening for the same participants. To avoid including the same individuals and outcomes more than once in the same analysis model, results for categories “all”, “men”, and “women” are limited to the largest study sample, which was evident at the first screening when participants were aged under 64. The analytical approach is shown in Figure 1.

Appendix Figure 5. Hazard ratios for incident cardiovascular disease per two-unit improvement in SCORE and ASCVD algorithm over 5-years stratified by age and sex from model including only those who achieved the change without medication.

Risk score	Population	Hazard ratio per 2-unit improvement in risk score (95% confidence interval)	p-value
**SCORE**	All	0·80 (0·72 to 0·89)	<0·0001
	Men	0·83 (0·74 to 0·93)	0·0013
	Women	0·73 (0·55 to 0·97)	0·0320
	40 to <50	0·78 (0·66 to 0·93)	0·0062
	50 to <60	0·79 (0·68 to 0·93)	0·0044
	60 to <70	0·82 (0·72 to 0·93)	0·0018
	70 to <75	1·03 (0·78 to 1·35)	0·8430
**ASCVD**	All	0·88 (0·85 to 0·92)	<0·0001
	Men	0·90 (0·86 to 0·94)	<0·0001
	Women	0·81 (0·69 to 0·95)	0·0088
	40 to <50	0·82 (0·75 to 0·90)	<0·0001
	50 to <60	0·91 (0·86 to 0·97)	0·0019
	60 to <70	0·93 (0·87 to 0·99)	0·0326
	70 to <75	0·93 (0·79 to 1·11)	0·4322

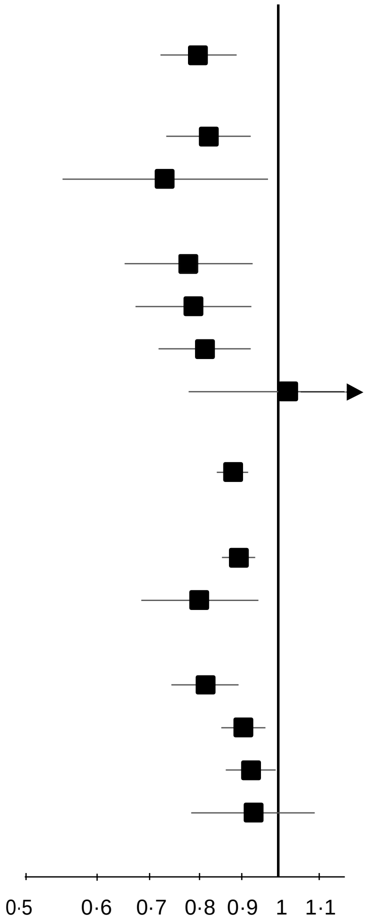

*All the analyses were adjusted for socioeconomic status, ethnicity, and baseline risk. Whitehall II included repeated screening for the same participants. To avoid including the same individuals and outcomes more than once in the same analysis model, results for categories “all”, “men”, and “women” are limited to the largest study sample, which was evident at the first screening when participants were aged under 64. The analytical approach is shown in Figure 1.

Appendix Figure 6. Hazard ratios for incident cardiovascular disease per two-unit improvement in SCORE and ASCVD algorithm over 5-years stratified by age and sex from model including competing risk of death.

Risk score	Population	Hazard ratio per 2-unit improvement in risk score (95% confidence interval)	p-value
**SCORE**	All	0·79 (0·72 to 0·87)	<0·0001
	Men	0·83 (0·76 to 0·91)	0·0001
	Women	0·67 (0·52 to 0·88)	0·0034
	40 to <50	0·76 (0·63 to 0·91)	0·0030
	50 to <60	0·85 (0·75 to 0·96)	0·0087
	60 to <70	0·93 (0·87 to 1.00)	0·0450
	70 to <75	0·94 (0·83 to 1·07)	0·3551
**ASCVD**	All	0·88 (0·85 to 0·92)	<0·0001
	Men	0·91 (0·88 to 0·95)	<0·0001
	Women	0·75 (0·70 to 0·80)	<0·0001
	40 to <50	0·81 (0·74 to 0·89)	<0·0001
	50 to <60	0·94 (0·86 to 1·03)	0·1885
	60 to <70	0·93 (0·90 to 0·97)	0·0004
	70 to <75	0·93 (0·88 to 0·98)	0·0106

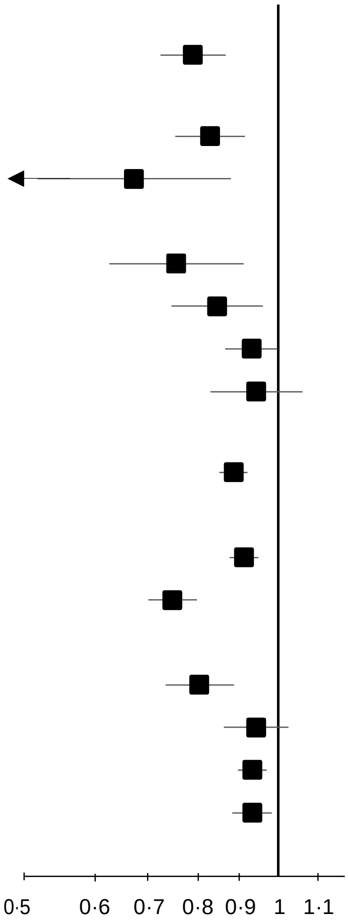

*All the analyses were adjusted for socioeconomic status, ethnicity, and baseline risk. Whitehall II included repeated screening for the same participants. To avoid including the same individuals and outcomes more than once in the same analysis model, results for categories "all", "men", and "women" are limited to the largest study sample, which was evident at the first screening when participants were aged under 64. The analytical approach is shown in Figure 1.

Appendix Figure 7. Hazard ratios for incident cardiovascular disease per two-unit improvement in SCORE and ASCVD algorithm over 5-years stratified by age and sex from multiple imputation analysis.

Risk score	Population	Hazard ratio per 2-unit improvement in risk score (95% confidence interval)	p-value
**SCORE**	All	0·79 (0·73 to 0·85)	<0·0001
	Men	0·83 (0·76 to 0·90)	<0·0001
	Women	0·67 (0·53 to 0·84)	0·0006
	40 to <50	0·77 (0·66 to 0·90)	0·0011
	50 to <60	0·85 (0·75 to 0·96)	0·0101
	60 to <70	0·93 (0·87 to 0·99)	0·0198
	70 to <75	0·94 (0·84 to 1·05)	0·2837
**ASCVD**	All	0·88 (0·85 to 0·91)	<0·0001
	Men	0·91 (0·87 to 0·94)	<0·0001
	Women	0·75 (0·69 to 0·80)	<0·0001
	40 to <50	0·80 (0·75 to 0·87)	<0·0001
	50 to <60	0·90 (0·86 to 0·94)	<0·0001
	60 to <70	0·94 (0·90 to 0·97)	0·0006
	70 to <75	0·93 (0·88 to 0·98)	0·0048

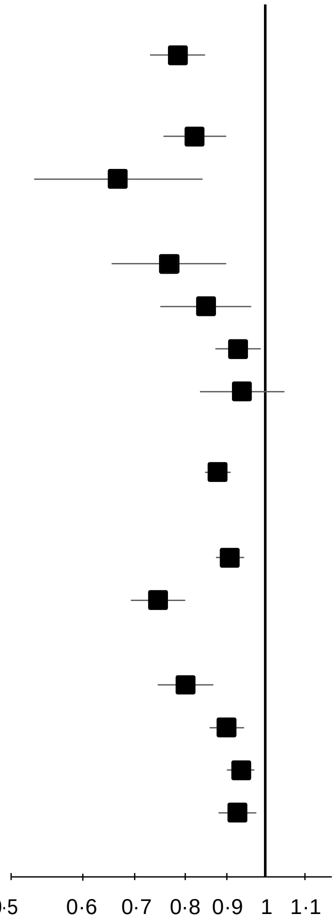

*All the analyses were adjusted for socioeconomic status, ethnicity, and baseline risk. Whitehall II included repeated screening for the same participants. To avoid including the same individuals and outcomes more than once in the same analysis model, results for categories “all”, “men”, and “women” are limited to the largest study sample, which was evident at the first screening when participants were aged under 64. The analytical approach is shown in Figure 1.

Appendix Figure 8. Hazard ratios for incident cardiovascular disease per two-unit improvement in SCORE and ASCVD algorithm over 5-years stratified by age and sex from model including only myocardial infarctions and strokes as outcome.

Risk score	Population	Hazard ratio per 2-unit improvement in risk score (95% confidence interval)	p-value
**SCORE**	All	0·74 (0·68 to 0·81)	<0·0001
	Men	0·78 (0·71 to 0·87)	<0·0001
	Women	0·58 (0·44 to 0·77)	0·0002
	40 to <50	0·74 (0·63 to 0·88)	0·0005
	50 to <60	0·77 (0·66 to 0·89)	0·0004
	60 to <70	0·93 (0·86 to 1·00)	0·0537
	70 to <75	0·94 (0·82 to 1·08)	0·4118
**ASCVD**	All	0·71 (0·64 to 0·79)	<0·0001
	Men	0·77 (0·69 to 0·86)	<0·0001
	Women	0·52 (0·43 to 0·63)	<0·0001
	40 to <50	0·55 (0·44 to 0·67)	<0·0001
	50 to <60	0·75 (0·66 to 0·87)	<0·0001
	60 to <70	0·95 (0·90 to 0·99)	0·0246
	70 to <75	0·97 (0·90 to 1·04)	0·3445

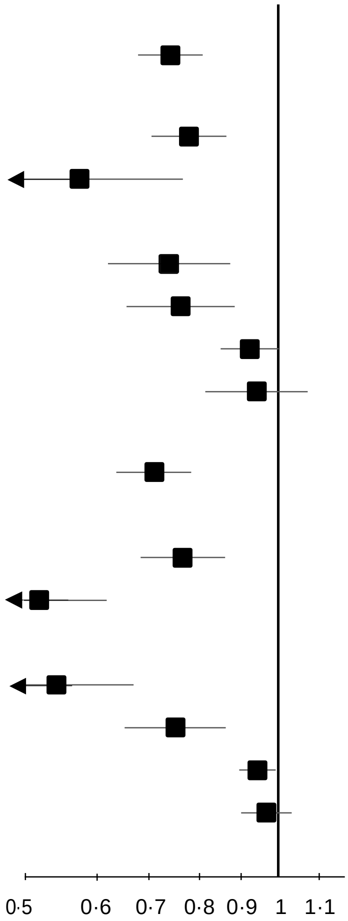

*All the analyses were adjusted for socioeconomic status, ethnicity, and baseline risk. Whitehall II included repeated screening for the same participants. To avoid including the same individuals and outcomes more than once in the same analysis model, results for categories “all”, “men”, and “women” are limited to the largest study sample, which was evident at the first screening when participants were aged under 64. The analytical approach is shown in Figure 1

Online tool and examples

The link for Online tool Github page is here:

https://github.com/ninamars/Change-in-CVD-risk-scores

Based on our results, we developed an interactive calculator to estimate cardiovascular disease-free life-years gained or lost based on preceding, current, and future risk factor measurements for SCORE or ASCVD risk scores at each age between 40 and 75. The preceding measurements (analysis of risk history) show how changes in risk factors between two health checks affect the risk score when compared to ageing only. The future measurements (analysis of targeted change) facilitate planning of targeted risk factor levels by quantifying the potential effects of future lifestyle change and medical interventions on risk factor levels.

As an example of analysis of risk history, consider a male smoker aged 55, with systolic blood pressure at 140mmHg, total cholesterol at 6mmol/l and HDL-C at 1mmol/l at his current measurement. When combined to data from earlier measurement when the participant was age 50, with systolic blood pressure at 130mmHg, total cholesterol at 5mmol/l and HDL-C at 1mmol/l, we can observe that his 10-year risk for cardiovascular diseases measured with SCORE has progressed from 1.8% to 4.3% over the 5-year period. The progression is faster than would have been observed with only changing age (risk with age only changing is 3%), and a loss of 1.3 diseases-free years is estimated. Risk of 4.3% would place the individual to moderate risk category where current ESC guidelines recommend considering medical intervention. Additional information on faster rate of risk progression could be considered as risk enhancing factor and support initiation of preventive medication.

The second example illustrate how the calculator can be used to facilitate planning of targeted risk factor levels by quantifying the potential effects of future lifestyle change and medical interventions on risk factor levels. Consider a 50 years old non-smoker male, with systolic blood pressure at 145mmHg, total cholesterol at 6mmol/l and HDL-C at 1mmol/l. Measured with ASCVD score the participant has 6.9% 10-year risk of cardiovascular diseases, which indicates consideration of preventive medication. However, the participant prefers lifestyle change to medication and decides to start DASH-diet, three sessions of aerobic endurance per week, and weight management to lose 5kg during 1 year. This would translate to:

**Intervention**	**Change in systolic blood pressure (mmHg) by baseline blood pressure**	**Change in total cholesterol (mmol/l) by baseline cholesterol concentration**
Start DASH diet	-11.4	-0.2
Aerobic endurance training (3 sessions per week)	-8.3	-0.1
A 5-kilogram reduction in weight	-5.5	-0.5
**Total**	**-25.2**	**-0.8**

These interventions, if successful, would prolong diseases free life by 0.7 years and lead to a 4.5% risk in the follow-up visit next year, or a 6.3% risk in the next five years after taking into account ageing. As such, the targeted lifestyle change would be sufficient to postpone preventive medication.

Appendix References

1. Pencina MJ, D'Agostino RB S, D'Agostino RB,Jr, Vasan RS. Evaluating the added predictive ability of a new marker: from area under the ROC curve to reclassification and beyond. *Stat Med* 2008; 27(2): 157,72; discussion 207-12. doi:10.1002/sim.2929 [doi].

2. Pencina MJ, D'Agostino RB S, Steyerberg EW. Extensions of net reclassification improvement calculations to measure usefulness of new biomarkers. *Stat Med* 2011; 30(1): 11-21. doi:10.1002/sim.4085 [doi].

3. Collins GS, Reitsma JB, Altman DG, Moons KG. Transparent reporting of a multivariable prediction model for individual prognosis or diagnosis (TRIPOD): the TRIPOD statement. *BMJ* 2015; 350: g7594. doi:10.1136/bmj.g7594 [doi].

4. Hastie T, Tibshirani R, Friedman J. The Elements of Statistical Learning Data Mining, Inference, and Prediction. 2nd ed. Springer; 2017.

5. Cox DR. Regression Models and Life-Tables. *Journal of the Royal Statistical Society Series B (Methodological)* 1972; 34(2): 187-220.

6. Akaike H. Information theory and an extension of the maximum likelihood principle. *Second International Symposium on Information Theory* 1973; ed. B. N. Petrov and F. Csaki, 267–281. Budapest: Akademiai Kiado.

7. Sterne JA, White IR, Carlin JB, et al. Multiple imputation for missing data in epidemiological and clinical research: potential and pitfalls. *BMJ* 2009; 338: b2393. doi:10.1136/bmj.b2393 [doi].

8. Lindbohm JV, Sipila PN, Mars NJ, et al. 5-Year Versus Risk-Category-Specific Screening Intervals for Cardiovascular Disease Prevention: a Cohort Study. *Lancet Public Health* 2019; 4(4): e189-99. doi:S2468-2667(19)30023-4 [pii].

9. Royston P, Parmar MK. Restricted mean survival time: an alternative to the hazard ratio for the design and analysis of randomized trials with a time-to-event outcome. *BMC Med Res Methodol* 2013; 13: 152,2288-13-152. doi:10.1186/1471-2288-13-152 [doi].

10. White IR, Royston P, Wood AM. Multiple imputation using chained equations: Issues and guidance for practice. *Stat Med* 2011; 30(4): 377-99. doi:10.1002/sim.4067 [doi].

11. Barnard J, Rubin DB. Small-Sample Degrees of Freedom with Multiple Imputation. *Biometrika* 1999; 86(4): 948–55.

12. Fine JP GR. A Proportional Hazards Model for the Subdistribution of a Competing Risk. *Journal of the American Statistical Association* 1999; (94): 496-509.

13. Eckel RH, Jakicic JM, Ard JD, et al. 2013 AHA/ACC guideline on lifestyle management to reduce cardiovascular risk: a report of the American College of Cardiology/American Heart Association Task Force on Practice Guidelines. *J Am Coll Cardiol* 2014; 63(25 Pt B): 2960-84. doi:10.1016/j.jacc.2013.11.003 [doi].

14. Arnett DK, Blumenthal RS, Albert MA, et al. 2019 ACC/AHA Guideline on the Primary Prevention of Cardiovascular Disease: A Report of the American College of Cardiology/American Heart Association Task Force on Clinical Practice Guidelines. *Circulation* 2019; 140(11): e596-646. doi:10.1161/CIR.0000000000000678 [doi].

15. Mach F, Baigent C, Catapano AL, et al. 2019 ESC/EAS Guidelines for the management of dyslipidaemias: lipid modification to reduce cardiovascular risk. *Eur Heart J* 2020; 41(1): 111-88. doi:10.1093/eurheartj/ehz455 [doi].

16. He FJ, Li J, Macgregor GA. Effect of longer term modest salt reduction on blood pressure: Cochrane systematic review and meta-analysis of randomised trials. *BMJ* 2013; 346: f1325. doi:10.1136/bmj.f1325 [doi].

17. Appel LJ, Moore TJ, Obarzanek E, et al. A clinical trial of the effects of dietary patterns on blood pressure. DASH Collaborative Research Group. *N Engl J Med* 1997; 336(16): 1117-24. doi:10.1056/NEJM199704173361601 [doi].

18. Aburto NJ, Hanson S, Gutierrez H, Hooper L, Elliott P, Cappuccio FP. Effect of increased potassium intake on cardiovascular risk factors and disease: systematic review and meta-analyses. *BMJ* 2013; 346: f1378. doi:10.1136/bmj.f1378 [doi].

19. Roerecke M, Kaczorowski J, Tobe SW, Gmel G, Hasan OSM, Rehm J. The effect of a reduction in alcohol consumption on blood pressure: a systematic review and meta-analysis. *Lancet Public Health* 2017; 2(2): e108-20. doi:S2468-2667(17)30003-8 [pii].

20. Domenech M, Roman P, Lapetra J, et al. Mediterranean diet reduces 24-hour ambulatory blood pressure, blood glucose, and lipids: one-year randomized, clinical trial. *Hypertension* 2014; 64(1): 69-76. doi:10.1161/HYPERTENSIONAHA.113.03353 [doi].

21. Hollaender PL, Ross AB, Kristensen M. Whole-grain and blood lipid changes in apparently healthy adults: a systematic review and meta-analysis of randomized controlled studies. *Am J Clin Nutr* 2015; 102(3): 556-72. doi:10.3945/ajcn.115.109165 [doi].

22. Berger S, Raman G, Vishwanathan R, Jacques PF, Johnson EJ. Dietary cholesterol and cardiovascular disease: a systematic review and meta-analysis. *Am J Clin Nutr* 2015; 102(2): 276-94. doi:10.3945/ajcn.114.100305 [doi].

23. Gylling H, Plat J, Turley S, et al. Plant sterols and plant stanols in the management of dyslipidaemia and prevention of cardiovascular disease. *Atherosclerosis* 2014; 232(2): 346-60. doi:10.1016/j.atherosclerosis.2013.11.043 [doi].

24. Mozaffarian D, Micha R, Wallace S. Effects on coronary heart disease of increasing polyunsaturated fat in place of saturated fat: a systematic review and meta-analysis of randomized controlled trials. *PLoS Med* 2010; 7(3): e1000252. doi:10.1371/journal.pmed.1000252 [doi].

25. Li Y, Jiang L, Jia Z, Xin W, Yang S, Yang Q, Wang L. A meta-analysis of red yeast rice: an effective and relatively safe alternative approach for dyslipidemia. *PLoS One* 2014; 9(6): e98611. doi:10.1371/journal.pone.0098611 [doi].

26. Patnode CD, Evans CV, Senger CA, Redmond N, Lin JS. Behavioral Counseling to Promote a Healthful Diet and Physical Activity for Cardiovascular Disease Prevention in Adults Without Known Cardiovascular Disease Risk Factors: Updated Evidence Report and Systematic Review for the US Preventive Services Task Force. *JAMA* 2017; 318(2): 175-93. doi:10.1001/jama.2017.3303 [doi].

27. Kelley GA, Kelley KS. Impact of progressive resistance training on lipids and lipoproteins in adults: a meta-analysis of randomized controlled trials. *Prev Med* 2009; 48(1): 9-19. doi:10.1016/j.ypmed.2008.10.010 [doi].

28. Cornelissen VA, Fagard RH, Coeckelberghs E, Vanhees L. Impact of resistance training on blood pressure and other cardiovascular risk factors: a meta-analysis of randomized, controlled trials. *Hypertension* 2011; 58(5): 950-8. doi:10.1161/HYPERTENSIONAHA.111.177071 [doi].

29. Cornelissen VA, Fagard RH. Effects of endurance training on blood pressure, blood pressure-regulating mechanisms, and cardiovascular risk factors. *Hypertension* 2005; 46(4): 667-75. doi:01.HYP.0000184225.05629.51 [pii].

30. Cornelissen VA, Smart NA. Exercise training for blood pressure: a systematic review and meta-analysis. *J Am Heart Assoc* 2013; 2(1): e004473. doi:10.1161/JAHA.112.004473 [doi].

31. Zomer E, Gurusamy K, Leach R, et al. Interventions that cause weight loss and the impact on cardiovascular risk factors: a systematic review and meta-analysis. *Obes Rev* 2016; 17(10): 1001-11. doi:10.1111/obr.12433 [doi].

32. Williams B, Mancia G, Spiering W, et al. 2018 ESC/ESH Guidelines for the management of arterial hypertension. *Eur Heart J* 2018; 39(33): 3021-104. doi:10.1093/eurheartj/ehy339 [doi].
